# Age-associated imbalance in immune cell regeneration varies across individuals and arises from a distinct subset of stem cells

**DOI:** 10.1038/s41423-024-01225-y

**Published:** 2024-10-24

**Authors:** Anna Nogalska, Jiya Eerdeng, Samir Akre, Mary Vergel-Rodriguez, Yeachan Lee, Charles Bramlett, Adnan Y. Chowdhury, Bowen Wang, Colin G. Cess, Stacey D. Finley, Rong Lu

**Affiliations:** 1 Department of Stem Cell Biology and Regenerative Medicine, Eli and Edythe Broad Center for Regenerative Medicine and Stem Cell Research, Keck School of Medicine, Los Angeles, CA 90033 USA; 2 Alfred E. Mann Department of Biomedical Engineering, University of Southern California, Los Angeles, CA 90089 USA; 3 Department of Quantitative and Computational Biology, University of Southern California, Los Angeles, CA 90089 USA

**Keywords:** aging, clonal tracking, hematopoietic stem cells, lineage bias, myelopoiesis, Myelopoiesis, Lymphopoiesis

## Abstract

The age-associated decline in immunity manifests as imbalanced adaptive and innate immune cells, which originate from the aging of the stem cells that sustain their regeneration. Aging variation across individuals is well recognized, but its mechanism remains unclear. Here, we used high-throughput single-cell technologies to compare mice of the same chronological age that exhibited early or delayed immune aging phenotypes. We found that some hematopoietic stem cells (HSCs) in early aging mice upregulated genes related to aging, myeloid differentiation, and stem cell proliferation. Delayed aging was instead associated with genes involved in stem cell regulation and the response to external signals. These molecular changes align with shifts in HSC function. We found that the lineage biases of 30% to 40% of the HSC clones shifted with age. Moreover, their lineage biases shifted in opposite directions in mice exhibiting an early or delayed aging phenotype. In early aging mice, the HSC lineage bias shifted toward the myeloid lineage, driving the aging phenotype. In delayed aging mice, HSC lineage bias shifted toward the lymphoid lineage, effectively counteracting aging progression. Furthermore, the anti-aging HSC clones did not increase lymphoid production but instead decreased myeloid production. Additionally, we systematically quantified the frequency of various changes in HSC differentiation and their roles in driving the immune aging phenotype. Taken together, our findings suggest that temporal variation in the aging of immune cell regeneration among individuals primarily arises from differences in the myelopoiesis of a distinct subset of HSCs. Therefore, interventions to delay aging may be possible by targeting a subset of stem cells.

## Introduction

Aging in the immune system is characterized by an excess of innate immune cells and a shortage of adaptive immune cells [1–5]. This aging phenotype is highly relevant to disease genesis, as elderly individuals often suffer from increased myeloid leukemia [3, 6, 7] and immune deficiencies [3, 6, 8, 9]. While all living organisms age, aging phenotypes manifest at different chronological ages among individuals [10, 11]. However, the cellular and molecular mechanisms underlying temporal aging variations across individuals remain unclear. Understanding the basis of this variation can provide insights into the triggers of age-associated physiological decline.

Stem cells maintain tissue homeostasis and replenish aged and damaged cells throughout the lifetime of an organism [12–14]. Previous studies have shown that the age-associated imbalance in immune cell abundance arises from the aging of hematopoietic stem cells (HSCs) [2, 6, 8, 15–17]. With age, HSCs undergo multiple functional changes, such as increased self-renewal, increased myelopoiesis, and decreased lymphopoiesis [2, 3, 6]. These impairments in stem cell functions are associated with molecular changes, including DNA damage, epigenetic remodeling, translation defects, and alterations in extracellular signaling [12–14, 18].

Several models have been proposed for HSC aging, where HSC clones — an HSC and all its progeny, including HSCs and differentiated cells — undergo various changes as the organism ages. In some models, clonal expansion is thought to play an important role [1, 19] and is monitored in clinical settings as an indicator of leukemia genesis in elderly individuals [20–24]. Conversely, other models suggest that clonal exhaustion and cell senescence drive age-associated functional decline in the hematopoietic and immune systems [6, 25–27]. In light of the functional heterogeneity of individual HSCs [28–30], it has also been proposed that immune aging results from shifts in the relative proportions of HSC subtypes that exhibit distinct lineage biases and may not necessarily involve intrinsic alterations to HSCs [2, 30].

During aging, individual HSC clones within an organism may accumulate different molecular alterations and develop heterogeneous functional changes. The onset of the immune aging phenotype is determined by the aggregated changes in all HSC clones within an organism. Just as variation in aging exists across individuals, we hypothesize that individual HSC clones also age heterogeneously and that specific changes to a subset of HSC clones may play a major role in driving the onset of an organism’s aging phenotype. Recent advances in clonal tracking, high-throughput sequencing, and single-cell transcriptome profiling techniques have enabled precise quantification of stem cell activities in vivo [29, 31, 32]. Here, we utilized these techniques to compare individual HSCs from mice of the same chronological age, genetic background, and housing conditions that exhibited or lacked an immune aging phenotype. While aging impacts the immune system and HSCs across multiple parameters, our study specifically focused on the ratio of B cells to granulocytes in the peripheral blood (BG ratio), as they are the most abundant adaptive and innate immune cells, respectively. This is motivated by previous studies in humans that linked the neutrophil-to-lymphocyte ratio to disease progression in various medical conditions, such as ischemic stroke, cerebral hemorrhage, major cardiac events, sepsis and infectious diseases, and multiple types of cancer [33, 34]. We show how individual HSC clones heterogeneously change their gene expression and immune cell regeneration with age. Some of these changes are strongly associated with the tissue-level aging phenotype. Our results reconcile several prevalent stem cell aging models by quantifying their respective contributions to the aging phenotype. Overall, these findings suggest that the aging of immune cell regeneration is driven primarily by the modulation of myelopoiesis in a subset of HSCs.

## Results

### The onset of immune aging varies across individual mice

**Fig. 1:**
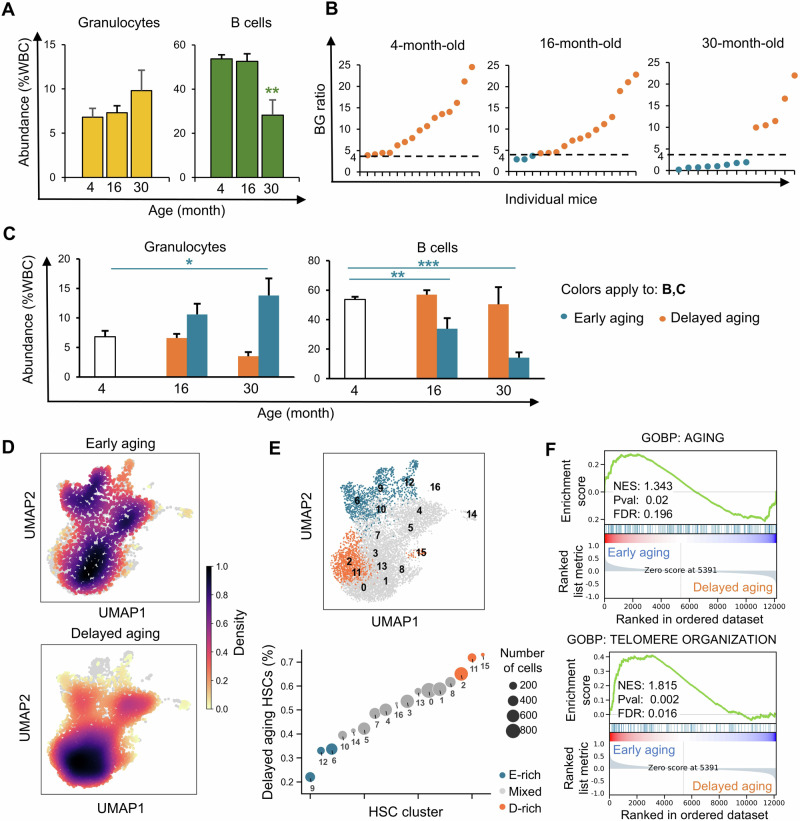
**The onset of immune aging varies across individual mice**. **A** Granulocyte and B-cell abundances in the peripheral blood of 4-, 16- and 30-month-old mice (*n* = 15, 16 and 13, respectively). **B** Individual mice from **A** are ranked on the basis of their ratios of B-cell abundance to granulocyte (Gr) abundance. The dashed line marks the lower boundary of the ratios from 4-month-old mice, which is used to discriminate between early and delayed aging. **C** Granulocyte and B-cell abundances in the peripheral blood of early and delayed aging mice. **A, C** Data are presented as the means ± SEMs. *P < 0.05, **P < 0.01, ***P < 0.001 compared between the early and delayed aging groups by two-tailed Student’s *t* test; WBC, white blood cells. **D** UMAP density plot of HSCs from early  and delayed aging mice. Each dot represents one cell. **E** Unsupervised clustering of HSCs identified 17 clusters. The top 3 clusters most enriched by HSCs from early aging mice or those from delayed aging mice are highlighted in different colors. **F** Gene set enrichment analysis (GSEA) profiles showing significant enrichment of genes associated with aging and telomere organization in the clusters overrepresented by HSCs from early aging mice. NES normalized enrichment score, FDR false discovery rate

To understand aging variation across individuals, we studied C57BL/6 J mice, the most widely used inbred strain. We measured the immune aging phenotype by calculating the BG ratio in the peripheral blood (Supplementary Fig. 1A). As expected, the mice generally presented increased levels of granulocytes and decreased levels of B cells at 30 months of age (Fig. 1A). However, we found substantial variations in the BG ratios across individuals (Fig. 1B). At 30 months of age, many mice presented BG ratios lower than those observed in 4-month-old mice. However, some 30-month-old mice maintained ratios similar to those of 4-month-old mice and thus lacked the expected aging phenotype (Fig. 1B). The variation across individuals was also evident at 16 months of age, when a smaller portion of individuals presented the aging phenotype (Fig. 1B).

Compared with 4-month-old mice, 30-month-old mice with reduced BG ratios had significantly greater levels of granulocytes and significantly lower levels of B cells (Fig. 1C). In contrast, 30-month-old mice with BG ratios similar to those of 4-month-old mice did not show significant shifts in B-cell or granulocyte abundance (Fig. 1C). Similar results were found when Mac1^+^ myeloid cells and T cells were analyzed (Supplementary Fig. 2). T cells were not extensively analyzed in this study because of their distinctive maturation and expansion in the thymus, as well as their distinct clonality in comparison to that of HSCs [35]. Our data demonstrate that variation exists in the onset of the immune aging phenotype among individual mice with the same genetic background and housing conditions. For subsequent analyses, we classified individual mice into two groups, the early and delayed aging groups, on the basis of whether their BG ratios at an advanced age fell below or within the range observed in mice at 4 months of age (Fig. 1B). There was no significant sex disparity between the early aging and delayed aging groups (Supplementary Table 1). By identifying elderly individuals exhibiting or lacking the immune aging phenotype at the same chronological age, we can investigate the mechanisms triggering the onset of the immune aging phenotype.

### Distinct subsets of HSCs in early and delayed aging mice differentially express aging-associated genes

To identify the gene expression signature associated with the variation in the onset of immune aging, we compared the transcriptomes of individual HSCs from 30-month-old mice exhibiting or lacking the aging phenotype (Supplementary Fig. 3A, Supplementary Table 2). While the transcriptomes of most HSCs were similar between the early and delayed aging groups (Fig. 1D), our unsupervised clustering analysis identified a few clusters that were overrepresented by HSCs from one group (Fig. 1E, Supplementary Fig. 3B to F, Supplementary Table 3). We refer to the top three clusters that were most enriched with early aging HSCs as early aging clusters and the top three clusters enriched with delayed aging HSCs as delayed aging clusters. A comparison of their gene expression profiles revealed significant overrepresentation of genes associated with aging and telomere organization in the early aging clusters (Fig. 1F). A comparison of the gene expression profiles of either early or delayed aging HSCs with those of HSCs from young mice revealed that the difference between early aging HSCs and young HSCs was substantially greater than the difference between delayed aging HSCs and young HSCs (Supplementary Fig. 4). This result indicates that among mice of the same chronological age, gene expression differences in a subset of HSCs align with the aging phenotype observed in immune cell counts in the peripheral blood.

### Distinct regulators of hematopoiesis are associated with early and delayed immune aging

**Fig. 2:**
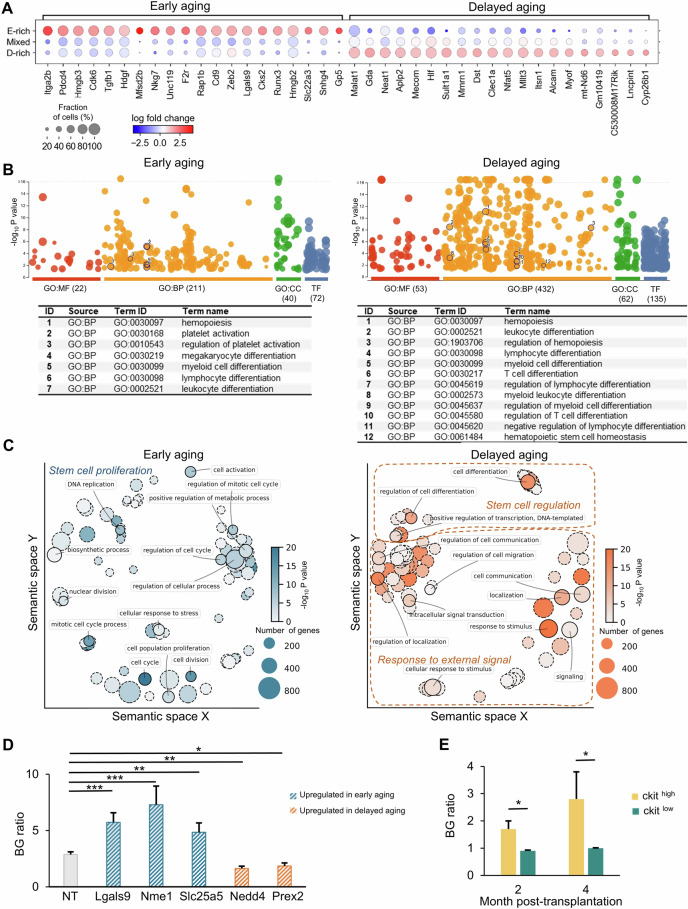
**Variation in the onset of immune aging is associated with distinct gene expression characteristics of HSCs.** **A** The top 20 DEGs in the clusters enriched with HSCs from early or delayed aging mice are shown (Fig. 1E). The dot size indicates the fraction of the HSCs expressing the gene. The color indicates the fold change in gene expression between the clusters of interest and all other clusters. **B** Gene Ontology enrichment and transcription factor binding motif analyses of upregulated genes in clusters enriched with HSCs from early and delayed aging mice. The circle size corresponds to the term size. The number of significantly enriched terms in each category is shown in parentheses. MF, molecular function; BP, biological process; CC, cellular component; TF, transcription factor. Selected terms related to hematopoiesis are listed in the table below. **C** Semantic similarity REViGO scatterplot of the top 100 GO:BP terms reveals that early aging is associated primarily with increased stem cell proliferation, whereas delayed aging is associated primarily with increased stem cell regulation and response to external signals. **D** Primary mouse HSCs from Cas9-expressing transgenic mice were transduced with lentivirus carrying a mixture of sgRNAs targeting each gene. A non-targeting (NT) sgRNA mixture was used as a control. The ratios of B cells (CD19^+^ B220^+^ ) to granulocytes (Mac1^+^ Gr1^+^) were analyzed after 10 days of co-culturing HSCs with OP9 cells. Two or more biological replicates with a total of 14 or more replicates per gene were performed. The data are shown as the means ± SEMs. Two-tailed Student’s *t* test. **E** BG ratio of hematopoietic cells in the peripheral blood derived from ckit high and ckit low donor HSCs. One-tailed Student’s *t* test was used. *P < 0.05; **P < 0.01; ***P < 0.001

Many genes upregulated in the early aging HSC clusters are involved in regulating myeloid differentiation (Supplementary Fig. 3G and Supplementary Data 1), including those of the top 20 most upregulated genes (Fig. 2A). In particular, *Itga2b* (CD41), the top-ranked upregulated gene, has been previously associated with HSC aging [36]. CD41-positive HSCs are known to exhibit myeloid bias [36] and increased expression of *Gata*1, which is also upregulated in the early aging HSC clusters (Supplementary Fig. 3G). Moreover, another upregulated gene, *TGF-β1*, has been shown to regulate HSC proliferation and stimulate myeloid-biased HSCs while inhibiting the growth of lymphoid-biased HSCs [37–39]. Additional regulators of myelopoiesis associated with early aging clusters include *Cd9* [40], *Hmgb2* [41], *Hmgb3* [42], and *Zeb2* [43]. The latter three genes, in addition to *Cdk6* [44], have also been shown to play roles in regulating HSC proliferation. Therefore, some of the genes whose expression was most upregulated in the early aging HSC clusters were associated with myeloid differentiation and HSC proliferation.

Among the top 20 most upregulated genes in the delayed aging clusters (Fig. 2A), many are transcriptional regulators, including *Hlf, Mecom, Nfat5*, and *Mllt3*, and long non-coding RNAs, including *Malat1*, *Neat1* and *Linc-pint* [45–51]. In particular, the long non-coding RNA *Linc-pint* is associated with healthy aging in mice, and knockout mice exhibit signs of premature aging in multiple tissues [52]. Additionally, activated leukocyte cell adhesion molecule (ALCAM) has been shown to counteract myeloid skewing, and HSCs in knockout mice exhibit aging-like phenotypes [53]. Therefore, some of the most upregulated genes in the delayed aging HSC clusters have already been shown to offset aging phenotypes.

### Variation in the onset of immune aging is associated with increased stem cell proliferation and decreased stem cell regulation through external signals

To comprehensively investigate the molecular mechanism underlying the variation in the onset of immune aging, we analyzed the biological implications of all genes that were significantly differentially expressed between the early and delayed aging clusters (Fig. 1E). We identified twice as many gene ontology (GO) terms and transcription factor binding motifs among genes expressed at significantly greater levels in the delayed aging clusters than in the early aging clusters (Fig. 2B and Supplementary Data 2). We focused on terms relevant to hematopoiesis and found that both the early and delayed aging clusters presented upregulated genes associated with GO terms related to hematopoiesis, myeloid cell differentiation, and lymphocyte differentiation, since these GO terms do not distinguish between positive and negative regulation. However, we also found substantial differences. For example, the early aging clusters were particularly enriched for genes associated with megakaryocyte differentiation and the regulation of platelet activation, whereas the delayed aging HSC clusters were uniquely enriched for genes associated with the regulation of T-cell differentiation and hematopoietic stem cell homeostasis (Fig. 2B).

Overall, genes upregulated in the early aging clusters were enriched in biological processes related to the stress response and stem cell proliferation, such as the regulation of the cell cycle (Fig. 2C). Genes that were upregulated in the delayed aging clusters were enriched in biological processes related to stem cell regulation and response to external signaling, such as cell communication and localization (Fig. 2C), both of which are fundamental for stem cell regulation. Taken together, these results suggest that the onset of immune aging is associated with increased stem cell proliferation and reduced control over stem cell homeostasis and differentiation, particularly in response to external signaling.

### Functional roles of DEGs between early aging and delayed aging HSC clusters

To investigate the functional roles of genes significantly differentially expressed between the early and delayed aging clusters, we performed CRISPR knockout assays using primary mouse HSCs co-cultured with OP9 cells to facilitate both myeloid and lymphoid differentiation. This analysis was conducted on genes with undefined roles in hematopoiesis. Our results revealed that knocking out genes upregulated in early aging clusters, such as *Lgals9*, *Nme1*, and *Slc25a5* (Supplementary Fig. 3H), led to an increase in the BG ratio (Fig. 2D), indicating that these genes contribute to reducing the BG ratio during aging. Conversely, knocking out genes upregulated in the delayed aging clusters, including *Nedd4* and *Prex2* (Supplementary Fig. 3I), resulted in a decreased BG ratio (Fig. 2D), suggesting their roles in maintaining the BG ratio in delayed aging mice. Additionally, ckit was significantly upregulated in delayed aging mice (Supplementary Fig. 3I). Therefore, we transplanted HSCs expressing ckit at high and low levels and found that the BG ratio derived from ckit^high^ HSCs was significantly greater than that derived from ckit^low^ HSCs (Fig. 2E). These in vitro and in vivo functional assays collectively demonstrated that genes differentially expressed in early and delayed aging HSC clusters play crucial roles in regulating immune cell production.

### Tracking individual HSC clones in early and delayed aging mice

**Fig. 3:**
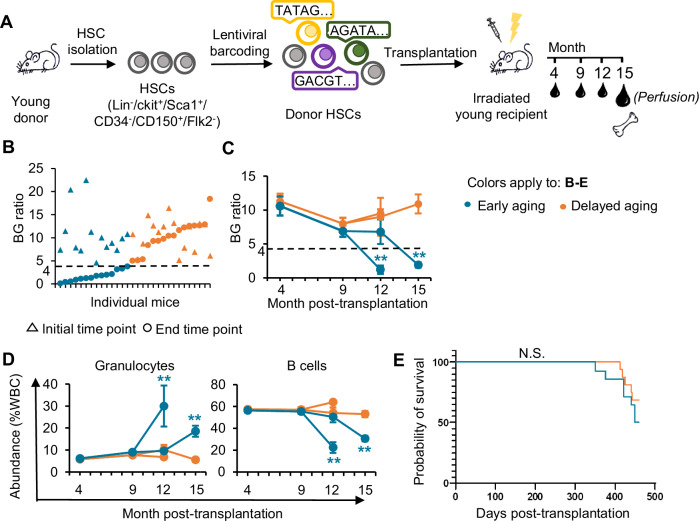
**Tracking individual HSC clones in early and delayed aging mice.** **A** Experimental design for tracking the immune cell production of individual hematopoietic stem cell (HSC) clones during aging. **B** Individual mice (*n* = 30) are ranked on the basis of their ratios of B-cell abundance to granulocyte (Gr) abundance (BG ratios) at the end time point. The dashed line marks the lower boundary of the corresponding ratios at the initial time point, which is used to discriminate between early and delayed aging. **C** Changes in the BG ratio at the population level over time in early (*n* = 13) and delayed aging (*n* = 12) mice. The dashed line is the same as in **B**. **D** Granulocyte and B-cell abundances in the peripheral blood over time. **C, D** One-way ANOVA with the Bonferroni-corrected paired *t* test was used to compare different time points; **P < 0.01 between the initial and end time points. Some mice died prior to 15 months post-transplantation, and their data at 12 months are shown separately to illustrate their end time points. **E** Kaplan‒Meier survival plots of early and delayed aging mice. **C, D** Data are presented as the means ± SEMs; N.S. not significant, WBC white blood cells

Since transcriptomic analysis revealed that differences in immune aging between early aging and delayed aging mice reside in a subset of HSCs (Figs. 1D–F, and 2), we further investigated how a subset of HSCs triggers the immune aging phenotype of an organism. To identify and characterize the HSC clones that drive the immune aging phenotype, we compared the immune cell production of individual HSC clones before and after the onset of immune aging. We isolated and labeled HSCs from young donor mice via unique genetic barcodes [29, 54] and transplanted them into young recipients (Fig. 3A, Supplementary Fig. 5 and Supplementary Table 1). The peripheral blood of recipient mice was analyzed starting from an initial time point 4 months post-transplantation, when hematopoiesis had stabilized at the cell population level [55, 56], to an end time point 15 months post -transplantation or 12 months post- transplantation if the mouse reached its end-of-life before the 15-month mark (Fig. 3B–D and Supplementary Fig. 5A–F). Similar to our analysis of naïve mice (Fig. 1), we classified individual mice into either the early or delayed aging group on the basis of their BG ratios at the end time point compared with the range of observed ratios for all mice at the initial time point (Fig. 3B and Supplementary Table 2). Mice in the delayed aging group may also develop the aging phenotype given additional time (Supplementary Fig. 5).

The immune aging phenotype of the mice in the early aging group started to develop after 9 months post-transplantation (Fig. 3C, D). Thus, we refer to 9 months post-transplantation as the pre-divergent time point. Like naïve non-transplanted mice (Fig. 1C), early aging mice had significantly higher levels of granulocytes and significantly lower levels of B cells at the end time point than at the initial time point, whereas delayed aging mice did not exhibit any substantial changes in granulocyte or B-cell abundance over time (Fig. 3D). Moreover, delayed aging mice tended to live longer than early aging mice (Fig. 3E). Compared with those in the delayed aging group (31.8%), almost twice as many mice in the early aging group (59%) became sick or died prior to the predetermined end point of the experiment. These findings indicate a correlation between mouse lifespan and the onset of immune aging, as delineated by the BG ratio in our analysis.

### Delayed onset of aging is associated with reduced myeloid production in a subset of HSC clones

**Fig. 4:**
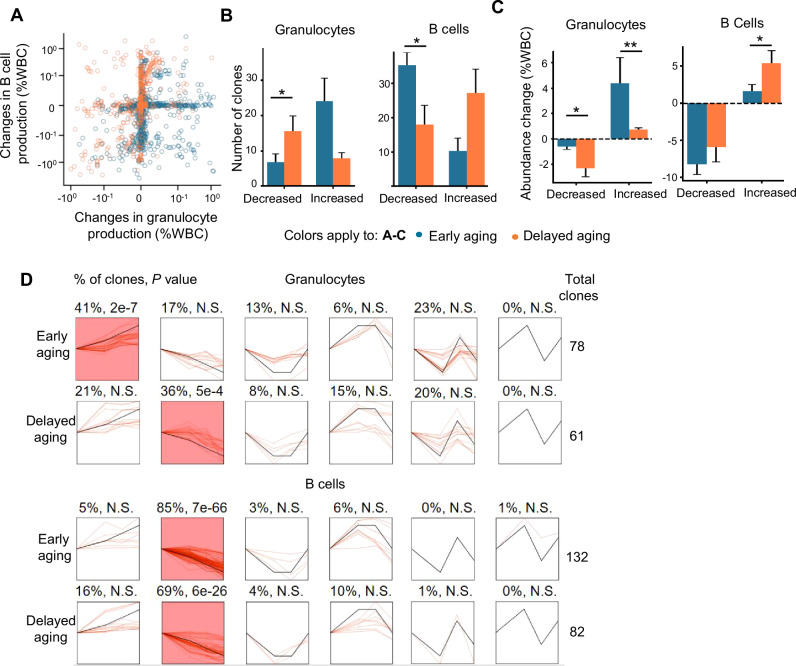
**The delayed onset of immune aging is associated with reduced myeloid production**. **A** Changes in B-cell and granulocyte production of individual HSC clones between the pre-divergent (9 months post-transplantation) and the end time points. Each circle indicates one HSC clone. Both axes are shown on a “symlog” scale with a linear scale ranging between 0 and 0.1. **B** The number of HSC clones that changed granulocyte or B-cell production in early and delayed aging mice. **C** Changes in the overall immune cell production of HSC clones that exhibited distinct changes in each mouse. **B, C** Clones with changes of less than 0.1% white blood cells were not considered. Bonferroni adjusted Wilcoxon rank-sum test. *P < 0.05, **P < 0.01. **D** STEM clustering [70] of HSC clones on the basis of changes in granulocyte and B-cell production over time. Each box shows a cluster of HSC clones, depicted as red lines, that share similar temporal dynamics, depicted as a black line. The colored boxes highlight clusters consisting of significantly more HSC clones than expected. The numbers above each box indicate the percentage of all clones in the corresponding cluster followed by the P value, indicating that the cluster consists of a greater number of clones than expected. N.S. not significant where *P* > 0.05. **B, C** Data are presented as the mean ± SEM; WBC white blood cells

To identify the specific changes in HSC clones that drive the immune aging phenotype, we compared their immune cell production at the pre-divergent time point, right before the onset of the aging phenotype, and at the end time point, when approximately half of the mice displayed the aging phenotype (Fig. 3B–D). Between the pre-divergent and end time points, the immune cell production of most HSC clones remained unchanged (Fig. 4A). The HSC clones whose immune cell production changed exhibited different characteristics between early aging and delayed aging mice. For example, the number of HSC clones that reduced B-cell production was significantly greater in early aging mice than in delayed aging mice (Fig. 4B). Among the HSC clones that increased B-cell production, the overall increase in B-cell abundance was significantly greater in delayed aging mice than in early aging mice (Fig. 4C). In the myeloid lineage, the number of clones with reduced granulocyte production was significantly greater in delayed aging mice than in early aging mice (Fig. 4B). The amount of overall granulocyte reduction was significantly greater in delayed aging mice, and the amount of overall granulocyte increase was significantly greater in early aging mice (Fig. 4C). Taken together, these data demonstrate differences in immune cell production among distinct subsets of HSC clones when early and delayed aging mice are compared.

The decrease in granulocyte production was unexpected, as granulocyte abundance generally increases with age (Fig. 1A and Supplementary Fig. 5B). However, a trend toward granulocyte reduction was detected in delayed aging mice at the population level (Figs. 1C and 3D). Moreover, the abundance of common myeloid progenitors was also significantly lower in delayed aging mice, but not early aging mice, than in young mice (Supplementary Fig. 5H). In addition, we analyzed the dynamic changes of HSC clones in granulocyte and B-cell production over time and found that the most significantly enriched temporal dynamics in granulocyte production were increasing in early aging mice and decreasing in delayed aging mice (Fig. 4D). Taken together, these findings suggest a compelling and surprising connection between reduced myeloid production and aging delay.

### The onset of immune aging is associated with distinct shifts in the lineage preferences of a subset of HSC clones

**Fig. 5:**
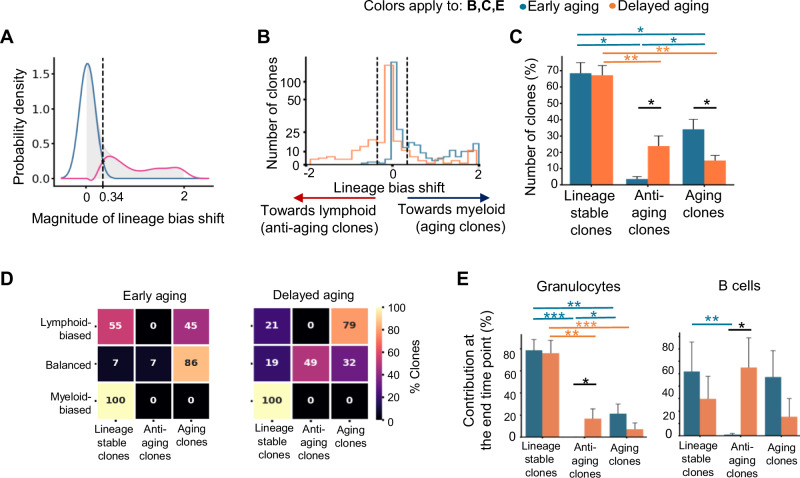
**The temporal variation of immune aging is associated with distinct changes to HSC lineage biases.** **A** Distribution of changes in the lineage bias of all HSC clones between the pre-divergent (9 months post-transplantation) and the end time points of the mice. A Gaussian kernel density estimate of the distribution is shown. The light gray area represents the distribution of all the clones. The blue curve shows the Gaussian fit for lineage stable clones. The remaining clones are highlighted by the pink curve. The black vertical dashed line shows the cutoff between lineage stable and lineage shifting clones that is set at the intersection of the black and pink curves. **B** Numbers of lineage stable and lineage shifting clones in the early and delayed aging groups. The dashed lines illustrate the thresholds that separate lineage stable and lineage shifting clones, as determined in **A**. Clone numbers are shown on a “symlog” scale with a linear scale ranging between 0 and 50. **C** Fractions of lineage stable, anti-aging and aging clones in early (*n* = 9) and delayed (*n* = 9) aging mice. **D** Heatmap showing the fraction of lymphoid-biased, myeloid-biased, and balanced clones whose lineage biases had shifted or remained stable by the end time point. The lineage bias category is determined by data from the pre-divergent time point. The median of all the mice in each group is shown. **E** Contribution of lineage stable and lineage  shifting clones to granulocytes and B cells in the peripheral blood at the end of the study. **C, E** Data are presented as the mean ± SEM; one-way ANOVA followed by Tukey’s HSD pairwise comparison within a group; Bonferroni-adjusted independent Student’s *t* test between groups. *P < 0.05, **P < 0.01, ***P < 0.001

Because immune aging manifests as concurrent changes in both myeloid and lymphoid lineages, we assessed the changes in lineage bias of HSC clones from the pre-divergent time point to the end time point. The aggregated data revealed that many HSC clones maintained their lineage bias during aging, forming a normal distribution centered around zero change (Fig. 5A). However, some clones exhibited changes in lineage bias to varying degrees (Fig. 5A). In all the mice, 60–70% of the HSC clones remained lineage stable (Fig. 5B, C). Among the HSC clones whose lineage preferences were altered, some shifted toward the myeloid lineage in line with the immune aging phenotype. Surprisingly, others shifted toward the lymphoid lineage and counteracted the aging process (Fig. 5B). We refer to the former HSC clones as “aging clones” and to the latter as “anti-aging clones”. Aging HSC clones were significantly more frequent in early aging mice, and anti- aging HSC clones were significantly more frequent in delayed aging mice (Fig. 5C). In addition, HSC clones with distinct lineage preferences presented different frequencies of lineage shifts. In particular, all the myeloid-biased HSC clones remained lineage stable, and all the anti-aging clones initially exhibited lineage balance (Fig. 5D).

To determine the functional significance of the lineage stable and lineage shifting HSC clones, we quantified their immune cell contributions. Our data revealed that 60–80% of myeloid cells were produced by lineage stable clones (Fig. 5E and Supplementary Fig. 6A), which aligns with the corresponding clone number (Fig. 5C). Surprisingly, lymphoid production depended on both lineage stable clones and lineage shifting clones to a similar degree (Fig. 5E and Supplementary Fig. 6A). These findings suggest that the small number of lineage shifting clones plays an important role in lymphoid production. In particular, anti-aging HSC clones produced substantial numbers of immune cells in both lineages in delayed aging mice, whereas their contribution to immune cell production in early aging mice was negligible (Fig. 5E and Supplementary Fig. 6A). These findings underscore the pivotal role of these clones in delaying the onset of the immune aging phenotype.

The discovery of aging-associated lineage shifting clones challenges the conventional paradigm that HSCs retain their differentiation characteristics and that daughter HSCs maintain their parental, epigenetically-defined lineage preferences [2, 57]. In addition, we found that the relative HSC abundances of myeloid-biased, balanced, and lymphoid-biased clones were similar between young and old mice and between early and delayed aging mice (Supplementary Fig. 6B), indicating that differential growth at the HSC level is not involved in triggering the immune aging phenotype. These data contradict the hypothesis that immune aging originates from shifts in the relative proportions of HSCs that exhibit different lineage biases [15, 58].

### Anti-aging clones shift their lineage bias toward the lymphoid lineage by reducing myelopoiesis

**Fig. 6:**
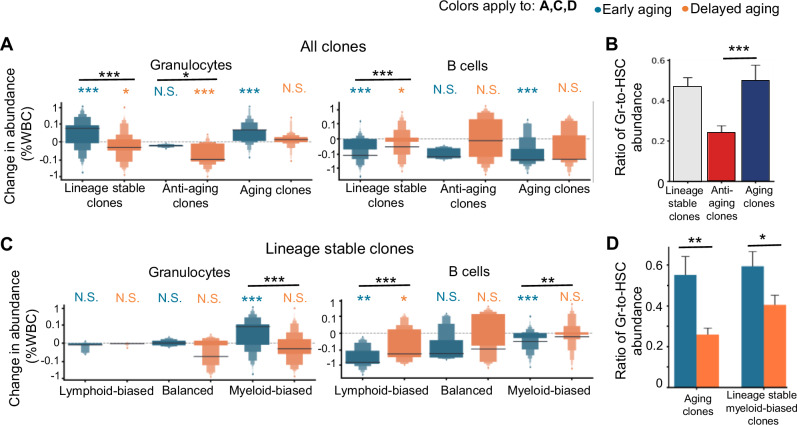
**Age-associated imbalance of the innate and adaptive immune systems is driven primarily by changes in myelopoiesis**. **A** Boxen plots showing changes in the immune cell production of HSC clones between the pre-divergent and end time points. **B** Quantification of the myeloid differentiation of lineage stable and lineage shifting HSC clones. **C** Boxen plots showing changes in immune cell production of lineage stable HSC clones between the pre-divergent and end time points. The lineage bias category is based on data from the end time point. **A, C** Each horizontal black bar denotes the mean of all the clones in each group. Independent Student’s *t* test was used for testing differences from 0; the Bonferroni-adjusted Wilcoxon rank-sum test was used between groups. **D** Quantification of the myeloid differentiation of aging clones and lineage stable myeloid-biased HSC clones. **B, D** Data are presented as the means ± SEMs; **B, D** Wilcoxon rank-sum test. Bonferroni correction was applied to **D**. *P < 0.05, **P < 0.01, ***P < 0.001. N.S. not significant, WBC white blood cells

Despite contributing significantly to B-cell production (Fig. 5E and Supplementary Fig. 6A), anti-aging HSC clones, on average, did not increase their B-cell production from the pre-divergent time point to the end time point (Fig. 6A). Instead, their myeloid production was significantly reduced, resulting in an apparent lineage bias toward the lymphoid lineage (Fig. 6A). A comparison of the granulocyte and HSC clonal abundances at the end time point revealed that anti-aging clones presented significantly lower levels of myeloid differentiation compared to other clones (Fig. 6B). The HSC abundance of these clones was not significantly different from that of the other clones (Supplementary Fig. 6C). These data suggest that the reduced myeloid production of the anti-aging HSC clones primarily arises from changes in HSC differentiation rather than self-renewal. This result challenges the clonal expansion aging model that was derived primarily from studies comparing young and old mice [6, 26, 59].

While most clones decreased their lymphoid production over time to various degrees, significant increases in myeloid production were detected in aging clones and lineage stable myeloid-biased clones from early aging mice (Fig. 6A, C). In contrast, the myeloid production of these clones did not increase with time in delayed aging mice (Fig. 6A, C). Moreover, these clones had significantly greater levels of myelopoiesis in early aging mice than in delayed aging mice when we compared granulocyte and HSC clonal abundance (Fig. 6D). However, their HSC abundances were not significantly different (Supplementary Fig. 6D, E). These results indicate that increased myeloid production with age primarily arises from changes in HSC differentiation rather than self-renewal. Taken together, our findings suggest that changes in myelopoiesis underlie immune aging.

### Clonal expansion, exhaustion, and activation in the aging of immune cell regeneration

**Fig. 7:**
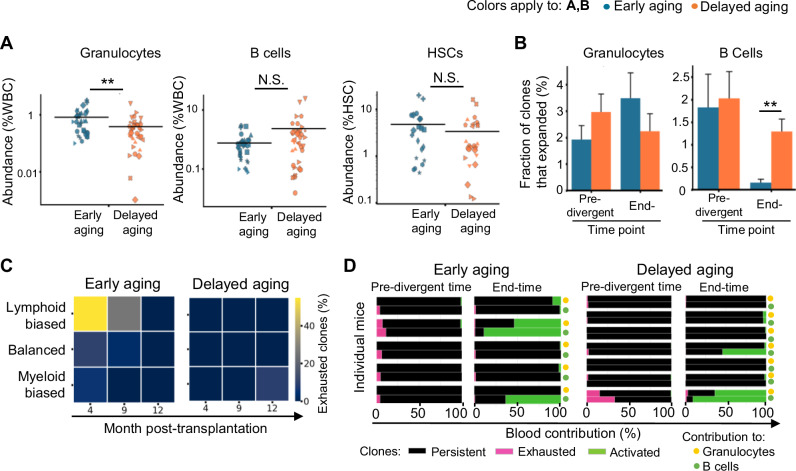
**Clonal expansion and exhaustion during aging**. **A** Abundance of the three most abundant clones in each mouse from the early (*n* = 21) and delayed aging (*n* = 27) groups at the end of the study. Each marker indicates one clone. Markers of a distinct shape represent data from the same mouse in each experiment in all figures. Each horizontal black bar denotes the mean of all the clones in each group. **B** Fractions of expanded clones in early (*n* = 9) and delayed aging (*n* = 9) mice. Expanded clones are those whose abundance is greater than a threshold defined by the lowest abundance of the most abundant clones whose aggregate abundance is at least half of all tracked clones at the pre-divergent time point. **A, B** Bonferroni-adjusted independent Student’s *t* test between groups. **C** Heatmap showing the fraction of lineage-biased and balanced clones that were exhausted by the end time point. The median of each group is shown. **D** Relative immune cell contributions of persistent, exhausted and activated clones in individual mice. Persistent clones are defined as clones that were present at both months 4 and 9 post-transplantation, as well as at the end time point. Activated clones are defined as clones that were absent at both months 4 and 9 but were present at the end time point. **C, D** Exhausted clones are defined as clones that were present at both months 4 and 9 post-transplantation but were not present at the end time point. **P < 0.01, N.S. not significant, WBC white blood cells

Clonal expansion and exhaustion are thought to play important roles in several aging models [1, 6, 19, 25, 26]. We quantified the abundance of the most abundant clones at the end time point and found that while early and delayed aging mice presented similar levels of clonal expansion at the HSC level, early aging mice presented a significantly greater degree of clonal expansion in granulocytes (Fig. 7A and Supplementary Fig. 6F–H). Moreover, early aging mice had significantly fewer expanded clones in producing B cells than delayed aging mice did (Fig. 7B). These data suggest that early aging is associated with a greater degree of clonal expansion during myelopoiesis and fewer expanded clones in the lymphoid lineage.

While the total number of HSC clones generating HSCs, granulocytes, and B cells was similar between early aging and delayed aging mice at all analyzed time points (Supplementary Fig. 5I–K), we found that HSC clones that initially displayed lymphoid biases were more prone to exhaustion with age in early aging mice but not in delayed aging mice (Fig. 7C). This implies that the early onset of aging is linked to the exhaustion of HSC clones that preferentially produce lymphocytes. However, the exhausted clones contributed only marginal amounts of immune cells before the onset of the aging phenotype in both early and delayed aging mice (Fig. 7D). In contrast to clonal exhaustion, we found that, in a few mice, substantial numbers of immune cells at the end time point were supplied by HSC clones whose immune cell contribution was undetectable at the initial and pre-divergent time points (Fig. 7D). We call this phenomenon clonal activation. Clonal activation was evident only in a small number of mice from both the early and delayed aging groups (Fig. 7D). Therefore, clonal exhaustion and activation do not appear to play major roles in distinguishing early and delayed aging phenotypes.

### Systematic comparison of various age-associated changes in immune cell regeneration

**Fig. 8:**
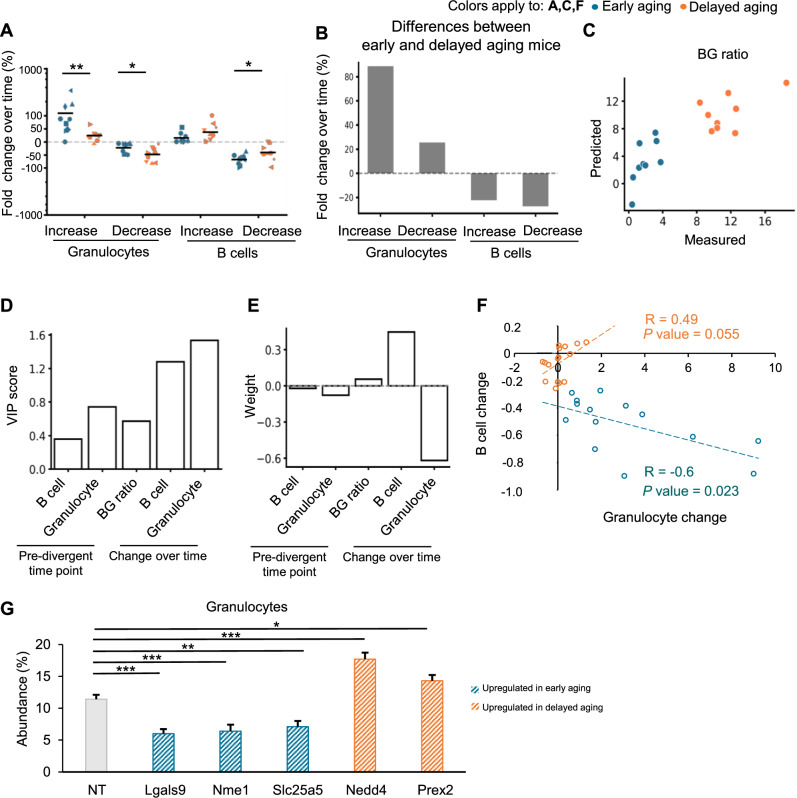
**Systematic comparison of various age-associated changes in immune cell regeneration**. **A** Changes in the production of different types of immune cells between the pre-divergent and end time points in early (*n* = 9) and delayed (*n* = 9) aging mice. Each marker indicates one mouse; each horizontal black bar denotes the mean of all the mice in each group. The change is shown on a “symlog” scale with a linear scale ranging between 0 and 100. Bonferroni adjusted Wilcoxon rank-sum test. **B** Differences in the average change in immune cell production between early aging and delayed aging mice, as shown in **A**. **C** A partial least squares regression (PLSR) model was used to determine the factors influencing the population-level B-cell-to-granulocyte ratio at the end of the study. A comparison of the predicted PLSR model outputs and the experimental measurements is shown. **D** Variable importance of projection (VIP) scores generated by the PLSR model. Generally, a VIP score greater than one means that the input is highly influential on the output. The pre-divergent and end-time-point ratios were determined at the cell population level via FACS analyses. The abundance of each lineage and their changes were determined on the basis of clonal-level measurements. **E** The final model weights are shown to illustrate the amount of positive or negative influence of each parameter. **F** Pearson’s correlation (R) between the normalized changes in population-level granulocyte and B-cell abundances in the peripheral blood. The normalized changes were calculated as the differences in abundances between the initial and the end time points divided by the abundances at the initial time point. **G** Primary mouse HSCs from Cas9-expressing transgenic mice were transduced with lentivirus carrying a mixture of sgRNAs targeting each gene. A non-targeting (NT) sgRNA mixture was used as a control. The abundance of granulocytes (Mac1^+^ Gr1^+^ ) among all living cells after 10 days of co-culturing HSCs with OP9 cells is shown. Two or more biological replicates with a total of 14 or more replicates for each gene were performed. The data are shown as the means ± SEMs. Two-tailed Student’s *t* test. *P < 0.05, **P < 0.01, ***P < 0.001

The immune aging phenotype can arise from four categories of changes in immune cell production: increasing myeloid production, decreasing lymphoid production, suppressing decreases in myeloid production, and suppressing increases in lymphoid production. To assess their respective impacts on the initiation of the aging phenotype, we compared the aggregate changes of all clones that underwent each type of change between the pre-divergent and end time points. In all four categories of changes, clones from early aging mice contributed more to the aging phenotype than those from delayed aging mice did (Fig. 8A, B). The predominant difference between early and delayed aging mice was their increase in myeloid production (Fig. 8A, B). These findings indicate that modulating the increase in myelopoiesis plays a pivotal role in triggering or delaying the onset of the immune aging phenotype.

To identify the factors influencing the tissue-level BG ratio at the end time point, we performed a partial least squares regression (PLSR) analysis (Fig. 8C–E). We found that the levels of lymphoid abundance, myeloid abundance, and their ratio at the pre-divergent time point all had negligible influences. Instead, changes in lymphoid and myeloid abundances were the dominant factors in determining the final BG ratio. In particular, changes in myeloid abundance appeared to be the most influential.

Among individual early aging mice, changes in the abundance of B cells and granulocytes were negatively correlated with each other (Fig. 8F), suggesting that their changes were synchronized. In contrast to the negative correlation in early aging mice, most delayed aging mice presented a positive correlation between B-cell and granulocyte abundance (Fig. 8F). Strikingly, five delayed aging mice deviated from the positive correlation by exhibiting a reduction in B-cell abundance without apparent changes in granulocyte abundance (Fig. 8F). The presence of these mice in delayed aging group indicates that a reduction in lymphoid cells alone does not trigger the immune aging phenotype. Instead, the increase in myeloid cells plays a key role in driving the onset of aging.

To further illustrate this point, we quantified the abundance of granulocytes derived from HSCs with CRISPR knockouts of selected genes that were significantly differentially expressed between the early and delayed aging clusters (Supplementary Fig. 3H, I). Our results revealed that knocking out genes upregulated in the early aging clusters, such as *Lgals9*, *Nme1*, and *Slc25a5* (Supplementary Fig. 3H), led to reduced granulocyte production (Fig. 8G), which was associated with an increased BG ratio (Fig. 2D). Conversely, knocking out genes upregulated in the delayed aging clusters, including *Nedd4* and *Prex2* (Supplementary Fig. 3I), resulted in increased granulocyte production (Fig. 8G), together with a decreased BG ratio (Fig. 2D). These data demonstrate the importance of myelopoiesis regulation in modulating the BG ratio.

## Discussion

In this study, we provide a new perspective on the aging of immune cell regeneration by comparing mice of the same chronological age at the organism, tissue, cell, and molecular levels. At the organism level, we classified individual mice into early aging and delayed aging groups on the basis of their BG ratio, which allowed us to study the mechanisms underlying the onset of aging (Figs. 1, 3). At the tissue level, we revealed coordinated age-associated changes in the myeloid and lymphoid lineages in the peripheral blood and related these changes to small subsets of stem cells in the bone marrow (Figs. 1, 3–6, 8). At the cellular level, we tracked the immune cell production of individual stem cell clones during aging and quantified their contribution to the onset of the immune aging phenotype (Figs. 5–7). At the molecular level, we identified specific genes and molecular functions that are associated with the early and delayed aging phenotypes (Figs. 1, 2). Our molecular and cellular analyses of both naïve mice and transplanted mice suggest that temporal variations in the onset of the immune aging phenotype across individual mice are primarily defined by a small subset of stem cells and that changes in myelopoiesis play a predominant role in triggering or delaying the onset of the immune aging phenotype. These findings provide insights into the heterogeneous immune aging phenotypes among individual mice of the same age and genetic background. In particular, our study advances the understanding of the myeloid bias shift in HSC aging through several key findings: (1) the myeloid bias shift of HSCs does not occur uniformly across all mice at the same age (Figs. 1, 3); (2) within each mouse, the myeloid bias shift occurs only in a small subset of HSC clones when the myeloid bias phenotype is detected in the peripheral blood (Fig. 5); and (3) some HSC clones can shift their lineage bias toward the lymphoid lineage with age, potentially delaying the onset of the aging phenotype (Fig. 5).

Previous aging studies have compared mostly young and old individuals due to limitations in technical sensitivity. Using state-of-the-art single-cell technologies such as single-cell RNA sequencing (Figs. 1, 2) and single-cell clone in vivo tracking (Figs. 3–8), we were able to identify differences in small subsets of stem cells from mice at the same chronological age that exhibited or lacked an immune aging phenotype. Understanding the mechanisms underlying the variation in the onset of an aging phenotype across individuals can help develop new therapeutic strategies for improving longevity and quality of life. While a few rejuvenation strategies have been proposed [14, 60, 61], delaying the onset of aging may be a more attainable goal than stopping or reversing the aging process. Our study revealed that delays in immune aging are associated with the suppression of myelopoiesis (Figs. 3–8), indicating that restraining myelopoiesis may be an effective therapy for delaying immune aging. We also identified specific subsets of stem cells and genes underlying the onset of immune aging (Figs. 1–6), providing potential cellular and molecular targets for therapeutic interventions to delay aging.

In addition to dissecting the heterogeneous aging of individuals, our study also elucidates the heterogeneous aging of individual stem cells. While most aging studies have investigated bulk cell populations, here, we show that specific subsets of HSCs in early and delayed aging mice exhibited significant age-associated differences in gene expression (Figs. 1, 2) and immune cell regeneration (Figs. 3–8). In particular, we identified a small subset of “anti-aging” stem cells in mice that exhibit a delayed aging phenotype and revealed their distinct gene expression and immune cell production characteristics (Figs. 1–8). Our results revealed that a subset of stem cells plays a dominant role in triggering the onset of the aging phenotype in the peripheral blood. Alterations in this HSC subset are sufficient to drive the aging phenotype, even when most HSCs remain unchanged. The heterogeneous aging of HSCs may be influenced by aging-associated changes in cytokines in the bone marrow, which could be explored in future studies. Our findings suggest that interventions to delay aging may be possible by targeting a small group of stem cells.

Finally, we compared various age-associated changes in stem cell functions by quantifying their frequency among stem cell clones and by evaluating their relative impact on the onset of immune aging (Figs. 3–8). We showed the quantitative contribution to the onset of aging from clonal expansion, exhaustion, and activation (Figs. 5–7) and from various changes in immune cell contributions (Figs. 4, 8). These data provide a comprehensive and systematic view of age-associated changes in the stem cell network. Our findings revealed how the heterogeneous aging of stem cell clones collectively determines physiological changes in an organism. We found that the predominant mechanism triggering the immune aging phenotype is myelopoiesis (Figs. 4–8). In particular, suppressing myelopoiesis in lineage-balanced stem cell clones is associated with delayed aging (Figs. 2–8). We identified several key regulators of myelopoiesis that may play a key role in this process (Fig. 2). These results reveal changes associated with the onset of the aging phenotype that can be targeted to manage aging through the development of new classes of therapeutic treatments. In summary, our research provides new insights into the variation in the onset of aging across individuals and identifies the cellular and molecular signatures associated with delays in the aging of immune cell regeneration. Our methodology of dissecting the heterogeneous aging of individual cell clones and comparing aging organisms of the same chronological age can be extended to study age-associated physiological changes in a wide variety of cells, tissues, organs, and organisms.

## Materials and methods

### Animals

The following mouse strains were used: C57BL/6J (CD45.2, Jackson Laboratory, stock #00664), B6.SJL-Ptprc^a^Pepc^*b*^/BoyJ (CD45.1, Jackson Laboratory, stock #002014), F1 (CD45.1/CD45.2, offspring of C57BL/6J and B6.SJL-Ptprc^a^Pepc^b^/BoyJ) (Supplementary Table 1). The donor and recipient mice were 8 to 12 weeks old at the time of transplantation. Recipient mice were irradiated with 950 cGy before receiving the transplant. The mice were bred and maintained at the University of Southern California Department of Animal Research facility. All animal procedures were approved by the Institutional Animal Care and Use Committee.

### HSC isolation and transplantation

HSCs (lineage (CD3, CD4, CD8, B220, Gr1, Mac1, Ter119)^−^/ckit^+^/Sca1^+^/Flk2^−^/CD34^−^/CD150^+^) were obtained from the crushed bones of donor mice and isolated via FACS sorting (Supplementary Fig. 1B) with a FACS-Aria II (BD Biosciences, San Jose, CA) after enrichment via CD117 microbeads (AutoMACS, Miltenyi Biotec, Auburn, CA). For each experiment, donor HSCs were obtained from at least two independent cohorts of young mice. FACS-sorted HSCs were infected with lentivirus carrying barcodes for 15 h before transplantation. HSC clonal labeling was performed as previously described [29, 54]. HSCs (3000–4500 per recipient) were transplanted via retro-orbital injection along with 250,000 whole bone marrow cells flushed from the femurs. ckit high and low HSCs were sorted (Supplementary Fig. 1C) from CD45.2 and CD45.1/CD45.2 donor mice and transplanted into busulfan-treated CD45.1 recipients at a 1:1 ratio. The end time point of the experiment was set at 15 months post-transplantation or 12 months post-transplantation if a mouse reached its end-of-life prior to the 15-month mark. To collect young HSCs, the mice were sacrificed at 6 months post-transplantation.

### Immune cell collection from the peripheral blood

Blood samples were collected from the tail vein into 10 mM EDTA-containing PBS, except when the mice were sacrificed. In the latter case, blood was collected via transcardial perfusion with 10 mM EDTA in PBS. After blood collection, 2% dextran was added to eliminate red blood cells, and the remaining blood cells were incubated with ammonium chloride-potassium lysis buffer on ice for 5 min to remove residual red blood cells. After 45 min of antibody incubation at 4 °C, the samples were suspended in PBS supplemented with 2% FBS and 4,6-diamidino-2-phenylindole to distinguish dead cells. The cells were sorted via a FACSAria II. Antibodies were obtained from Life Technologies (Carlsbad, CA) and BioLegend (San Diego, CA) as described previously [62, 63].

The following cell surface markers were used to sort immune cell populations (Supplementary Fig. 1A):

Granulocytes: CD4^−^/CD8^−^/B220^−^/CD19^−^/Mac1^+^/Gr1^+^/side scatter high.

B cells: CD4^−^/CD8^−^/Gr1^−^/Mac1^−^/B220^+^/CD19^+^.

CD4^+^ T cells: B220^−^/CD19^-^/Mac1^−^/Gr1^−^/TCRαβ^+^/CD4^+^/CD8^−^

CD8^+^ T cells: B220^−^/CD19^−^/Mac1^−^/Gr1^−^/TCRαβ^+^/CD4^−^/CD8^+^

Flow cytometry data were analyzed via BD FACSDiva software version 8.0 and FlowJo software version 10.4.2 (Tree Start, Ashland, OR).

### Bone marrow cell collection

At the end of the experiment, bone marrow was collected by crushing the bones, followed by enrichment with CD117 microbeads. The following cell surface markers were used to sort bone marrow hematopoietic progenitors (Supplementary Fig. 1D):

MPP^-^: lineage (TCR, CD4, CD8, B220, Gr1, Mac1, Ter119)^−^/cKit^+^/Sca1^+^/Flk2^−^/CD34^+^

MPP^+^ : lineage (TCR, CD4, CD8, B220, Gr1, Mac1, Ter119)^−^/cKit ^+^/Sca1^+^/Flk2^+^

CMP: lineage (TCR, CD4, CD8, B220, Gr1, Mac1, Ter119)^−^/cKit^+^/Sca1^−^/FcγR^−^/CD34^+^

GMP: lineage (TCR, CD4, CD8, B220, Gr1, Mac1, Ter119)^−^/cKit^+^/Sca1^−^/FcγR^+^/CD34^+^

MEP: lineage (TCR, CD4, CD8, B220, Gr1, Mac1, Ter119)^−^/cKit^+^/Sca1^−^/FcγR^−^/CD34^−^

CLP: lineage (TCR, CD4, CD8, B220, Gr1, Mac1, Ter119)^−^/Flk2^+^/IL7Rα^+^ 

Antibodies were obtained from Life Technologies (Carlsbad, CA) and BioLegend (San Diego, CA) as described previously [62, 63].

### Extraction and sequencing of DNA-tracking barcodes

Genomic DNA (gDNA) was extracted from cells via a Quick-gDNA MicroPrep kit (Zymo Research, Irvine, CA) according to the manufacturer’s instructions. gDNA was amplified via Phusion High-Fidelity PCR Master Mix (Thermo Scientific, Waltham, MA). PCRs were run on a ViiA7 RT‒PCR thermocycler (Life Technologies) with 0.2x Eva Green fluorescent dye (Biotium, Hayward, CA) to monitor DNA amplification. The reactions were stopped once they had progressed halfway through the exponential phase. The PCR products were purified via SPRIselect Beads (Beckman Coulter, Brea, CA) and quantified via a Qubit double-stranded DNA high-sensitivity assay kit (Invitrogen, Carlsbad, CA). The purified PCR products were analyzed via high-throughput sequencing via the Illumina NextSeq High v2 Kit (Illumina, San Diego, CA). Sequencing data were analyzed via custom Python code. The Python code and step-by-step protocol for barcode extraction were published previously [54].

### Single-cell RNA sequencing and analysis

For single-cell RNA sequencing (scRNA-seq), we FACS-sorted HSPCs, including myeloid progenitors [lineage (CD3, CD4, CD8, B220, Gr1, Mac1, Ter119)^−^/IL-7R^+^/Sca^−^, common lymphoid progenitors [lineage (CD3, CD4, CD8, B220, Gr1, Mac1, Ter119)^−^/IL7R^+^/Flk2^+^], and KLS cells [lineage (CD3, CD4, CD8, B220, Gr1, Mac1, Ter119)^−^/ckit^+^/Sca1^+^] (Supplementary Fig. 1D), from three early and three delayed aging mice that were 30 months old. Sorted cells were washed in PBS with 0.04% bovine serum albumin (BSA) and processed via the Chromium Single-cell 3′ v3.1 Library Kit (10× Genomics, Pleasanton, CA) following the manufacturer’s instructions. A total of 16,000 single cells were loaded for capture. Complementary DNA was synthesized and amplified for a total of 11 cycles. After quality checking and quantification via high-sensitivity DNA tape (Agilent, Santa Clara, CA), the cDNA libraries were sequenced via an Illumina HiSeq 3000/4000 kit at a coverage of 55,000 raw reads per cell (paired end; read 1:26 cycles; i7 index:8 cycles; read 2:98 cycles).

The sequencing data were first processed via the CellRanger pipeline (10x Genomics, v6). The “*cellranger count”* function identified a total of 54,559 cells from early and delayed aging mice. Scanpy (v1.9.1) [64] was used for the downstream data analyses unless specified otherwise. We excluded cells with more than 10% UMIs mapped to mitochondrial genes and cells with a total UMI count and gene count of more than three absolute deviations from the median of all cells. We also excluded cells whose total UMI was less than 1000 and whose total number of genes was less than 500. The cell types were annotated via SingleR (v1.10.0) [65] with reference data from the Gene Expression Common database (gexc.riken.jp, Mouse Hematopoiesis and Stroma model). The annotation returned 7,724 HSCs that were used in the analysis for Figs. 1D–E and 2. Batch effects were corrected via the Harmony algorithm [66]. For downstream analyses, only genes with at least 3 UMIs in at least 5% of the cells were used.

Differentially expressed genes (DEGs) were identified via ‘scanpy.tl.rank_gene_groups’ (with method = ‘Wilcoxon’ and corr_method = ‘Benjamini‒Hochberg’). Genes with adjusted *P* values < 0.05 and expressed in at least 20% of the cells were considered differentially expressed (DEGs). Gene set enrichment analysis (GSEA) [67] was performed with log-normalized gene expression data to compare early aging and delayed aging cells via GSEA software (http://software.broadinstitute.org/gsea/index.jsp, MsigDB v7.5.1). Gene sets from GO:BP Aging and GO:BP Telomere Organization were used to generate Fig. 2C and Supplementary Fig. 3. For the GO enrichment analysis in Fig. 2, differentially expressed genes with more than two-fold changes were subjected to g:Gost functional profiling via g:Profiler [68] (https://biit.cs.ut.ee/gprofiler/gost version e106eg53p1665fcd97) with the default settings. The top 100 returned GO:BP terms from the early and delayed aging groups were subsequently submitted to REViGO [69] (http://revigo.irb.hr) for semantic similarity analysis (Fig. 2C).

### Quantification of clonal abundance

Custom Python scripts were used to extract barcode information from the raw FASTQ files as previously described [29, 54]. The script is available through our previous publication [54]. The lentiviral vector that delivers clonal tracking barcodes also carries GFP, which marks the barcoded cells. To quantify the contribution of each HSC clone to each white blood cell type, we combined sequencing data with FACS data as follows:

$${Clonal\; abundance\; in\; the\; blood}\;\left( \% {WBC}\right) =\,	 \left({cell\; type}\;\% \;{in\; white\; blood\; cell}\right) \\ 	 \times \left({donor}\; \% \;{in\; the\; cell\; type}\right) \\ 	 \times \left({GFP} \; \% \; {among\; donor\; cells}\right) \\ 	 \times \left({number\; of\; reads\; for\; one\; barcode}\right) \\ 	 \times 100 \; \% \; /({total\; reads\; of\; all\; barcodes})$$

Clones with an abundance higher than 0.01% among white blood cells at least one time point were included in the analyses.

The HSC clonal abundance was calculated as follows:

$${Clonal\; abundance\; of\; HSCs}\;\left( \% {of\; all\; HSCs}\right) =\,	 \left({donor} \; \% \right) \times \left({GFP}\; \% {among\; donor\; cells}\right) \\ 	 \times \left({read\; number\; of\; one\; barcode}\right)\\ 	 \times 100 \;\% \,/\left({total\; reads\; of\; all\; barcodes}\right)$$

### Lineage bias analysis

To calculate lineage bias, first, the normalized abundance of each clone in an immune cell type was calculated by dividing the clone’s abundance by the corresponding cell population abundance (FACS data) at the pre-divergent time point for primary recipients. Next, a baseline lineage balance, theta ranging from 0 to pi/2, was calculated on the basis of the normalized abundance. Finally, lineage bias was calculated by converting theta values to the range [−1, 1], where 1 is myeloid committed and −1 is lymphoid committed.

$$\Theta = \arctan \left(\frac{{Gr\; normalized}}{B\;{normalized}}\right)$$

$${Lineage\; bias}=\left(2* \left(\theta -\frac{\pi }{4\,}\right)\right)$$

Lineage-balanced clones are defined as those whose theta falls between $\frac{\pi }{8}$ and $\frac{3\pi }{8}$, exclusively. Lymphoid-biased clones are defined as those whose theta falls between 0 and $\frac{\pi }{8}$, inclusive, and myeloid-biased clones are defined as those whose theta falls between $\frac{3\pi }{8}$ and $\frac{\pi }{2}$, inclusive.

Lineage bias is calculated only for the clones whose granulocyte or B-cell abundance is greater than or equal to the minimal threshold (Gr_min_ or B-cell_min_) in each mouse as follows.

$${{Gr}}_{\min }^{i}=\frac{{{{\rm{\#}}}}{initial\; Gr\; Cells}}{{{{\rm{\#}}}}{initial}\;B\;{cells}}\times {B}_{\min }$$

$${B}_{\min }=0.05$$

The lineage bias shift was calculated for each clone by subtracting its lineage bias at the pre-divergent time point from its lineage bias at the end time point. The distribution of the shifts in lineage bias (Fig. 5A) was generated by aggregating the absolute values of the lineage bias shift data from all the clones. The collective lineage bias shift dataset was then used to generate a Gaussian kernel density estimate (y_0_) (Fig. 5A). A Gaussian distribution curve was fitted around the highest peak in the kernel density estimate to identify lineage stable clones (y_unchanged_). The remaining clones were considered lineage shifting clones, y_changed_ (y_changed_ = y_0_ – y_unchanged_). The cutoff between lineage stable and lineage shifting clones was set at the first intersection of y_changed_ and y_unchanged._

### Clonal expansion, exhaustion and activation

To determine a threshold for clonal expansion, we ranked the abundance of all clones of one cell type at the pre-divergent time point. Among the clones with the highest abundances, the minimal number of clones whose accumulated abundances reached at least half of all the tracked clones were identified in each mouse. The mean of the lowest abundance of these clones from each mouse was used as the threshold for the clonal expansion of the corresponding cell type in all the mice. Exhausted clones are defined as clones that were present at both months 4 and 9 post-transplantation but not at the end time point. Activated clones are defined as clones that were absent at both months 4 and 9 but were present at the end time point. Persistent clones are defined as clones that were present at both months 4 and 9 post-transplantation, as well as at the end time point.

### Short time series expression miner (STEM)

STEM version 1.3.12 [70] was used to cluster and visualize dynamic changes in clonal abundance. Clones from mice with clonal data available at all 4 time points were included in this analysis. For each immune cell type, the clones were filtered such that the minimum change in abundance was 0.01% WBC between a later time point and the initial time point. STEM was executed with the normalization set to “log normalize”, the number of clusters set to six and default settings for the other parameters.

### Partial least squares regression (PLSR) model

The inputs for the PLSR model were (1) B-cell abundance at the pre-divergent time point determined by summing the data from individual clones in each mouse, (2) granulocyte abundance at the pre-divergent time point determined by summing the data from individual clones in each mouse, (3) the ratio of population-level B-cell abundance to granulocyte abundance at the predivergent time point from FACS measurements, (4) the net difference in B-cell abundance for individual clones between the pre-divergent time point and the end time point of a mouse, and (5) the net difference in granulocyte abundance for individual clones between the pre-divergent time point and the end time point of a mouse. The output for the PLSR model was the ratio of population-level B-cell abundance to granulocyte abundance at the end time point from FACS measurements. Each mouse represents one data point. Our final model consisted of two principal components. We calculated the variable importance of projection (VIP) scores via the principal component weights and output variance [71]. Generally, a VIP score greater than one means that the input is highly influential on the output [72]. To train the PLSR model, we used the nonlinear iterative partial least squares algorithm [73].

### In vitro lymphoid-myeloid differentiation assay

HSCs sorted via FACS (Supplementary Fig. 1B) from Cas9-expressing mice (B6J.129(Cg)-Gt(ROSA)26Sortm1.1(CAG-cas9*,EGFP)Fezh/J; Jackson Laboratory, #026179) were transduced with lentiviruses carrying a mixture of single-guide RNAs (sgRNAs) targeting each of the selected genes. Nongenome-targeting sgRNAs were used as negative controls. sgRNAs were designed and cloned as previously described [74] (Supplementary Table 4). The transduced HSCs were subsequently cultured on a layer of OP9 cells in αMEM with 10% FBS and 1% penicillin‒streptomycin (Life Technologies) supplemented with mSCF (50 ng/mL), mIL-7 (20 ng/ml), and mFlt3L (50 ng/ml) to facilitate their differentiation [75]. After 10 days of culture, the abundances of B cells (B220^+^CD19^+^) and granulocytes (Mac1^+^Gr1^+^) among all transduced (RFP^+^) cells were analyzed via flow cytometry (Supplementary Fig. 1E). For each gene, the experiment was performed at least twice, with *n* = 14 or more replicate wells per gene. The wells with fewer than 100 recorded granulocytes were excluded from the analysis. The transduced cells (RFP^+^) were also sorted and used for on-target editing verification via Sanger sequencing followed by indel analysis via the Inference of CRISPR Edits (ICE) from Synthego (https://www.synthego.com/products/bioinformatics/crispr-analysis) [76] (Supplementary Table 4).

### Statistical analysis

As described in the corresponding legends, the data are presented as the means ± SEMs (standard error of the mean), or values from individual mice or clones are shown with horizontal lines depicting the means. In the box and whisker plots, the box limits are the upper and lower quartiles, with the line indicating the median; the whiskers are the extremes, and the points are outliers. In the boxen (letter value) plot, the vertical line indicates the median, the innermost box represents the upper and lower fourths, an incrementally narrower box represents the lower and upper eighths, and an even narrower box represents the upper and lower sixteenths. A darker color indicates a higher data density. Points are outliers.

Unless specified otherwise, plots were generated via Microsoft Excel with the Python packages “matplotlib”, “seaborn” and “Scanpy”. All the mice with pertinent data available were included in the applicable analyses (Supplementary Table 2). Because it was not possible to collect data at all time points for every mouse, the number of mice used for different data analyses varied. The statistical methods used are indicated in the corresponding figure legends. The level of significance was set at *P* < 0.05, two tailed. Statistical analyses were performed in Excel or via Python scripts.

## Supplementary information

Supplementary Tables 1-4 and Supplementary Figures 1-6
