# Supplementary material for: Age-associated imbalance in immune cell regeneration varies across individuals and arises from a distinct subset of stem cells
Source: Cell Mol Immunol. 2024 Oct 24;21(12):1459–73. doi: 10.1038/s41423-024-01225-y (PMC11607082; doi:10.1038/s41423-024-01225-y)
Supplement: Supplementary file 1 — Supplementary Tables 1-4 and Supplementary Figures 1-6 [file 41423_2024_1225_MOESM1_ESM.pdf]

Supplementary Table 1

A

	Number of early aging mice	Number of delayed aging mice
**Gender**		
Female	4	1
Male	4	4

B

	Number of early aging mice	Number of delayed aging mice
**Donor strain**		
CD45.1	9	14
CD45.2	5	2
CD45.1/CD45.2	0	0
**Recipient strain**		
CD45.1	4	1
CD45.2	9	14
CD45.1/CD45.2	1	1
**Recipient gender**		
Female	9	8
Male	5	8

Supplementary Table 1. Characteristics of experimental mice. Early and delayed aging manifested similarly in mice of different strains and genders. (**A**) 30-month-old naïve non-transplanted mice. (**B**) mice that received HSC transplantation.

Supplementary Table 2

Figure	Number of mice included	Inclusion criteria
Fig. 1A-C, S2	4 -month-old - 15, 16 -month-old - 16, 30 -month-old - 13,	naïve mice available
Fig. 1D-F, 2A-C, S3, S4	early aging - 3, delayed aging - 3	old naïve mice with sc-RNAseq data
Fig. 3B, 8F	30	mice from multiple aging experiments that had blood data at the initial and the end time points
Fig. S5A-E	16	mice with blood data at 4 time points
Fig. S5G	1	a mouse observed until 17 months post transplantation
Fig. 3C,D,E S5F	early aging - 13, delayed aging - 12	mice with blood data at 4 and 3 time points
Fig. 4A-C, 5A-C, 6A-D, 7B, 8A-E	early aging - 9, delayed aging - 9	mice with barcoded blood data at 4 and 3 time points
Fig. 7C	early aging - 8, delayed aging - 9	mice with barcoded blood data at 4 and 3 time points including the initial time point
Fig. 7D	early aging - 5, delayed aging - 7	mice with more than 20% of the barcoded cells in both lineages and less than 80% of undefined clones
Fig. 4D, S5J, S6G	early aging - 4, delayed aging - 6	mice with barcoded blood data at 4 time points
Fig. S5K, S6G,H	early aging - 4, delayed aging - 5	mice with barcoded blood at 4 time points and HSC data
Fig. 5D,E, S6A	early aging - 6, delayed aging - 7	mice with more than 20% of the barcoded cells in both lineages
Fig.S6B, S5H	4	young mice with HSC data at month 6 post transplantation
Fig. 7A, S5I, S6F-G	early aging - 11, delayed aging - 13	mice with barcoded blood data at 4, 3 and 2 time points
Fig. S6B-E, S5G,H,	early aging - 7, delayed aging - 9	aged mice with barcoded HSC data at the end time point
S4	young - 3	young naïve mice with sc-RNAseq data
Fig. 2E	Ckit high/low co-transplantation - 9	recipients of ckit high/low HSCs

Supplementary Table 2. Inclusion criteria and numbers of mice used for data analyses. For each analysis, all mice with available data were used.

Supplementary Table 3

Cluster	Marker genes
0	Mllt3, Apoe, Nupr1, Sbspon, Procr
1	Rpl23, Rplp1, Rps3a1, Rpl13, Rps14, Rpl7, Rpl26
2	Neat1, Kcnq1ot1, Mecom, Dst, Angpt1, Meis1, Rock2
3	Hacd4, Nceh1, Prtn3, Pik3ip1, Msi2, Gm19590, Gcnt2
4	Tespa1, Serpinb1a, Satb1
5	Sox4, Ptpre, Arhgap30
6	Itga2b, Pf4, Rap1b, Pdcd4, Cd9, Pbx1, Tgfb1, Atpif1, Nrgn, Unc119
7	Vamp8, Mt1, Car2, Mif, Txn1, Rpl28, Ldha, Sdsl, Eif5a
8	Iigp1, Gm4951, Stat1, Gbp7, Igtp, Rtp4, Irgm1, Oasl2
9	Cdk6, Vim, Hmgb3, Slc22a3, Fam117a, Ptma, Gpx1
10	Mcm3, Mcm7, Dut, Stmn1, Mcm6, Hells, Mcm5, Mcm2, Lig1, Tipin
11	Pik3r1, Aplp2, Meg3, Alcam, Cyp26b1
12	Hmgb2, Ccnb2, H2afz, Hmgb1, Cenpa, H2afv, Cenpe, Knstrn, Mki67, Ptms
13	Nfkbia, Ltb, Junb, Serpina3g, Tnip3, Il2rg, Relb
14	Id2, Ccl5, Il2rb, Bcl2, Trbc1, Ptprc
15	H2-Eb1, H2-Ab1, Ctss, Lsp1, Irf8, Ighm
16	Tifa, Pde2a, Atp1b3, Crip1, Lgals9, Chchd10, B3gnt2, Pkig, Cd79b

Supplementary Table 3. Marker genes for each HSC cluster.

Supplementary Table 4

Gene	sgRNA sequence	KO score
Lgals9	CAACACCGCGTACCCTACCACCTCG	72
	CAGGGGTGCTGTGAACCATATGGA	
	CCACAGGAAACCACTTACATAGGCT	
Nme1	TGGTCTCTCCAAGCATCACG	41
	TGGTCTCTCCAAGCATCACG	
	TCCCAGGCTTAGAGTCTGCG	
Slc25a	CAAGGGCATCATAGACTGCG	56
	CTGCGTGGTTCGTATCCCCA	
	GCACAAGGATGTAGCCCCAG	
Nedd4	TAACCGGTAAAGGATAGAGA	26
	CCAGCATCCCCGACGAGCAG	
	CCAGCATCCCCGACGAGCAG	
Prex2	GAAGAATACAGACGTTCCCC	39
	GCTATCTAGCTGCACACCGA	
	CATATACAACCCCATACTCG	

Supplementary Table 4. sgRNA sequences and Synthego's ICE analysis results. The KO Score indicates the proportion of cells with detected indels that are likely to result in loss-of-function mutations.

Supplementary Figure 1

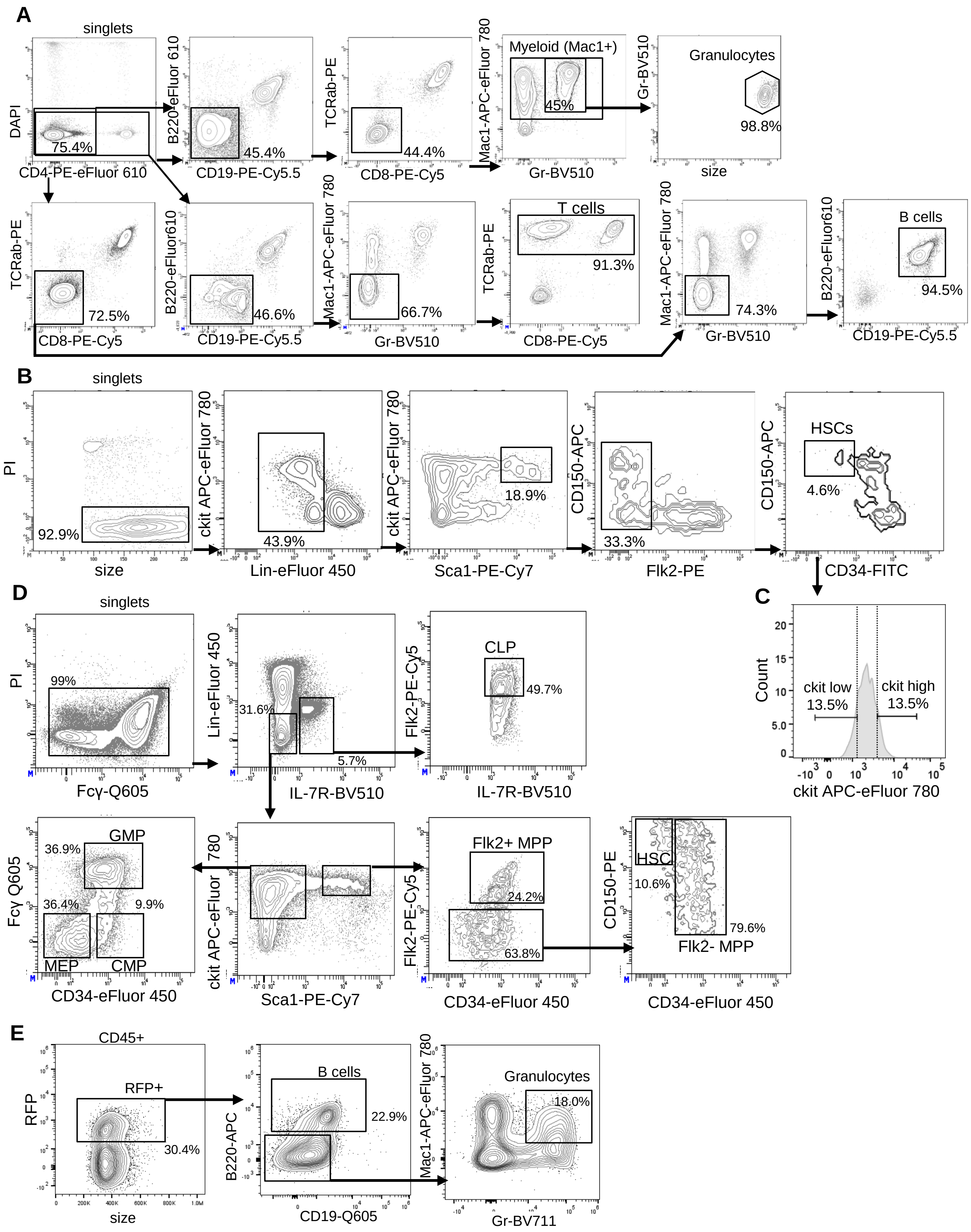

Supplementary Figure 1. Flow cytometry gating. (**A**) Flow cytometry gating for sorting granulocytes and B cells, and for analyzing Mac1+ myeloid cells and T cells from peripheral white blood cells. (**B**) Flow cytometry gating for sorting HSCs from c-Kit enriched bone marrow cells. (**C**) Flow cytometry gating for sorting HSCs and hematopoietic progenitor cells from c-Kit enriched bone marrow cells. (**D**) Flow cytometry gating for *in vitro* differentiation assay analysis.

Supplementary Figure 2

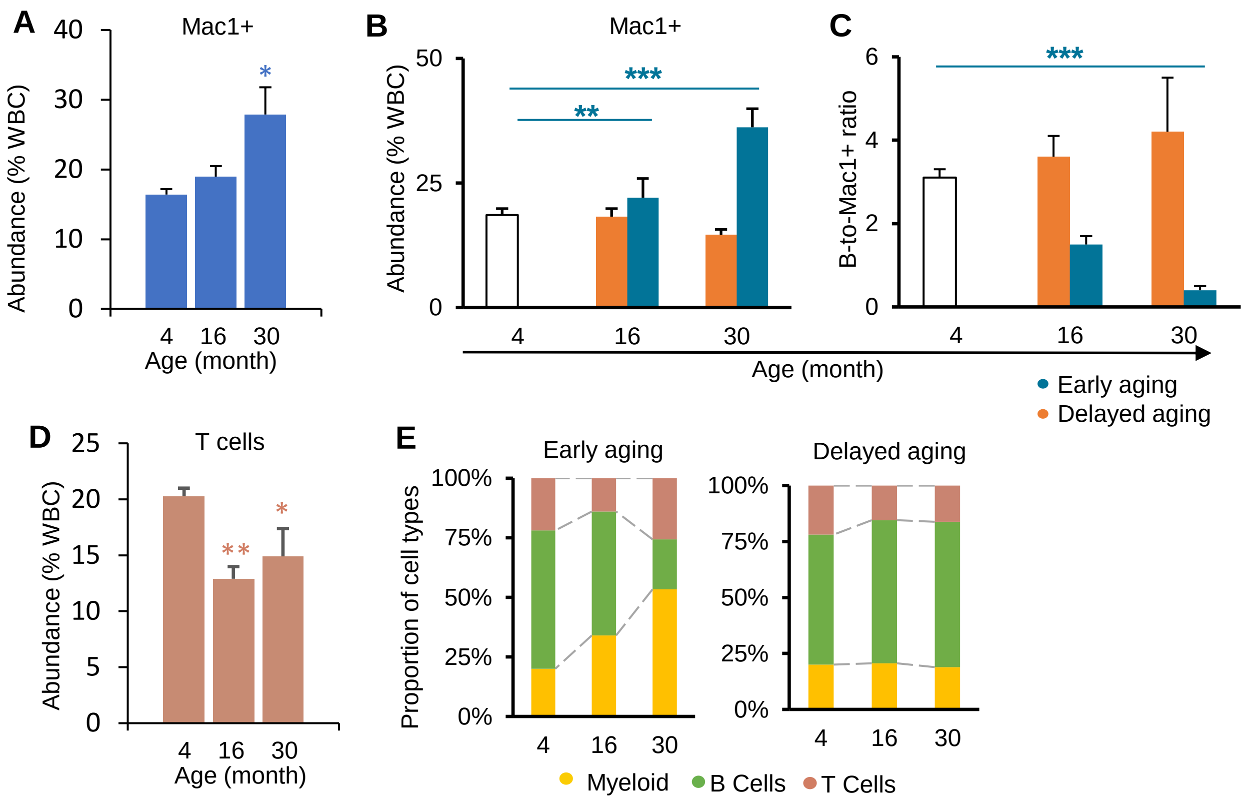

Supplementary Figure 2. Immune cell production in naïve aging mice. (**A**) Abundance of Mac1+ cells in the peripheral blood of 4-, 16- and 30-month-old mice (n = 15, 16 and 13 respectively). (**B**) Abundance of Mac1+ cells in the peripheral blood of early and delayed aging mice. (**C**) Change in the B cell to Mac1+ cell ratio at the population level over time in early and delayed aging mice. (**D**) Abundance of T cells in the peripheral blood of 4-, 16- and 30-month-old mice (n = 15, 16 and 13 respectively). (**E**) Myeloid (Mac1+), B and T cell abundance in the peripheral blood of early and delayed aging mice. (**A-D**) One-way ANOVA with Bonferroni post-hoc test comparing different time points; *P<0.05, **P<0.01, ***P<0.001 between initial and end time points.

Supplementary Figure 3

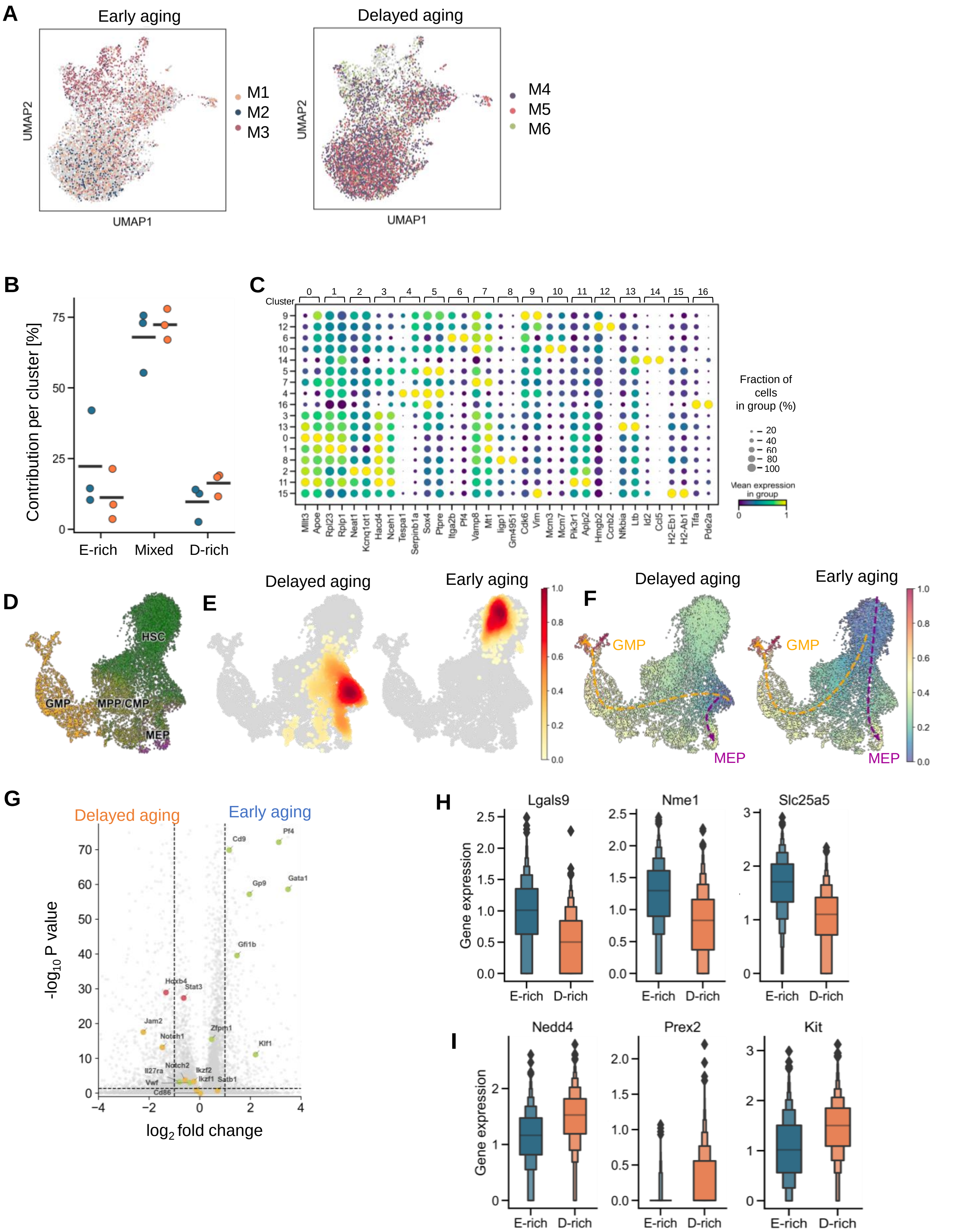

Supplementary Figure 3. Characterization of the early and delayed aging HSC clusters. (**A**) HSCs from individual mice in the early and delayed aging group overlayed on the UMAP plot of all HSCs (grey dots). Each dot represents one cell. (**B**) Contribution of each early and delayed aging mouse to the early and delayed aging HSC clusters. Each dot represents one mouse. (**C**) Expression levels of two representative marker genes for each HSC cluster. (**D**) UMAP plot illustrating HSCs, MPPs, CMPs, MEPs and GMPs from early and delayed aging mice. (**E**) UMAP density plot showing the distribution of early and delayed aging HSCs. (**F**) Developmental trajectories of early and delayed aging HSCs. (D-F) Each dot represents one cell. (**G**) Volcano plot showing differentially expressed genes in HSCs from early and delayed aging clusters (Fig. 2A). Highlighted are select myeloid-related genes (green), lymphoid-related genes (yellow) and genes involved in self-renewal (red). The vertical dashed lines delineate two-fold changes. The horizontal dashed line indicates P value of 0.05 with Benjamini-Hochberg correction. (**H**) Significant upregulation of Lgals9 (P value = 4.99e-95), Nme1 (P value =6.26e-26), and Slc25a5 (P value =6.91e-128) in early aging HSCs. (**I**) Significant upregulation of Nedd4 (P value =1.51e-58), Prex2 (P value =2.16e-33), and ckit (P value = 1.28e-54) in delayed aging HSCs.

Supplementary Figure 4

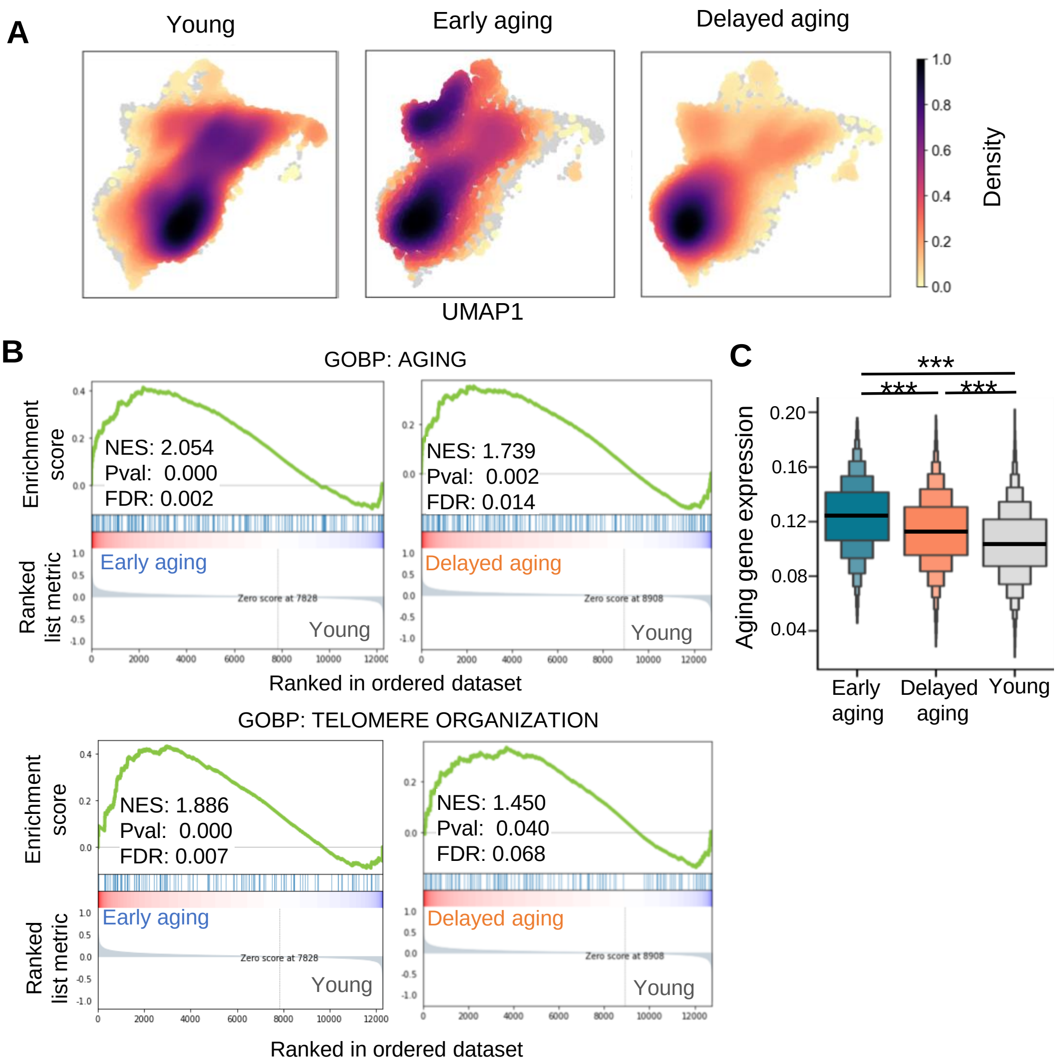

Supplementary Figure 4. Comparing gene expression of HSCs from young, early and delayed aging mice. (**A**) UMAP density plot of HSCs from young, early aging and delayed aging mice. (**B**) Gene Set Enrichment Analysis (GSEA) profiles show significant enrichment of genes associated with aging and telomere organization upregulated in the clusters overrepresented by HSCs from early or delayed aging mice when compared to young mice. NES, Normalized Enrichment Score; FDR, False Discovery Rate. (**C**) Comparing expression levels of aging-associated genes among young, early aging, and delayed aging HSCs. The genes analyzed are the same as in (**B**). ***P<0.001 Student's t-test with Benjamini-Hochberg correction.

Supplementary Figure 5

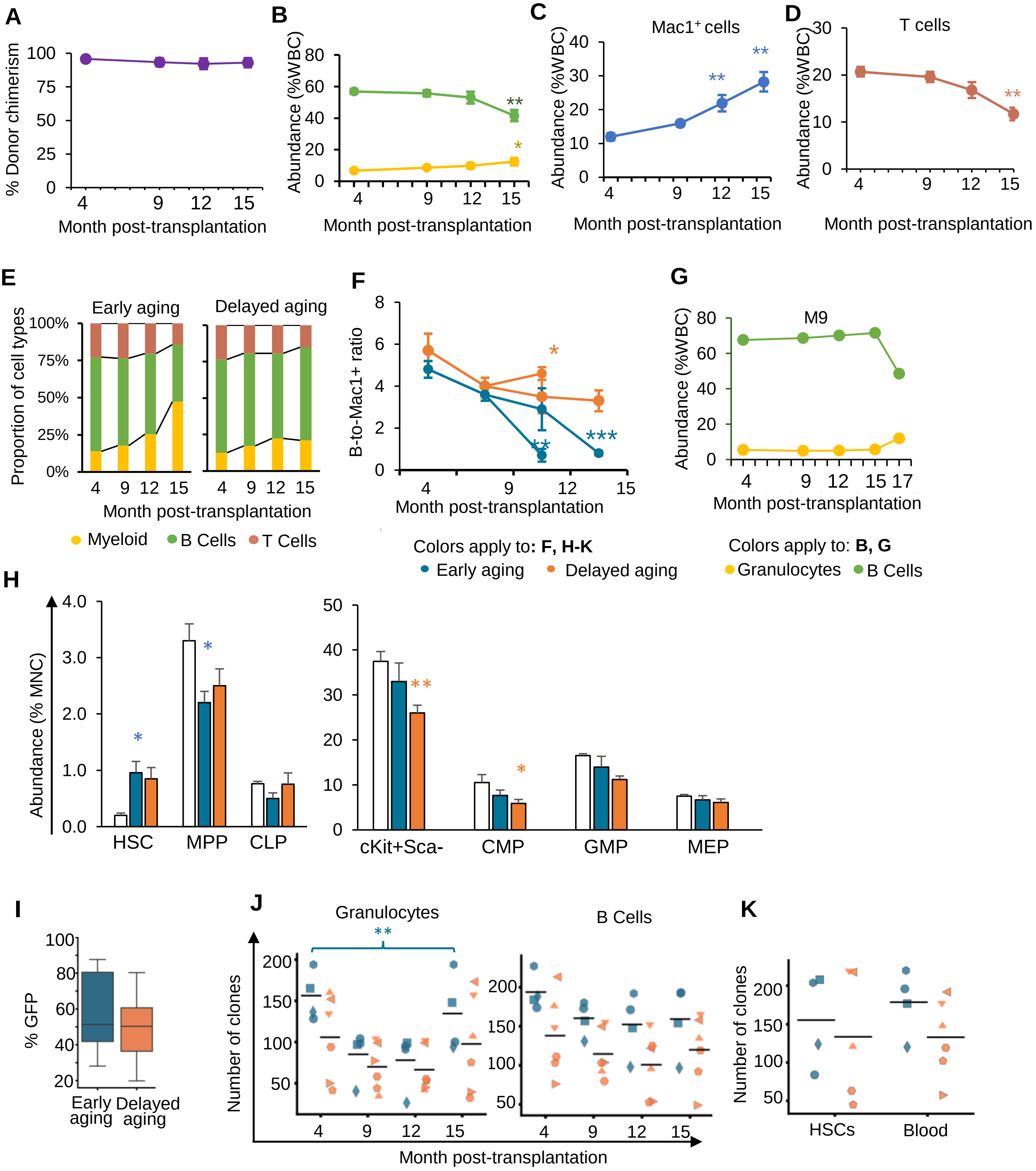

Supplementary Figure 5. Immune cell production in aging mice after transplantation. (**A**) Donor chimerism of granulocytes in the peripheral blood of recipient mice (n=16). (**B**) Total granulocyte and B cell abundance in the peripheral blood of early and delayed aging mice (n=16). Total Mac1+ (**C**) and T cells (**D**) abundance in the peripheral blood of early and delayed aging mice over time. (**E**) Myeloid (Mac1+), B and T cell abundance in the peripheral blood of early and delayed aging mice. (**F**) Change in the B cell to Mac1+ cell ratio at the population level over time in early and delayed aging mice. (**B,C,F**) One-way ANOVA with Bonferroni post-hoc test comparing different time points; *P<0.05, **P<0.01, ***P<0.01 between initial and end time points (**G**) Total granulocyte and B cell abundance in the peripheral blood of a mouse with an extended end time point 17 months post transplantation. (**H**) Comparison of HSPC abundance in the bone marrow of young (n=4), early (n=7) and delayed (n=9) aging mice at the end time point. MPP– multipotent progenitors, CLP – common lymphoid progenitors, CMP – common myeloid progenitors, GMP – granulocyte-monocyte progenitors, MEP – megakaryocyte-erythroid progenitors. *P<0.05, **P<0.01 as compared to young. One-way ANOVA with Bonferroni post-hoc test. (**I**) Fraction of granulocytes from the peripheral blood that express GFP, indicating transduction by barcoding lentivirus, among all donor-derived granulocytes at the initial time point. (**J**) Number of unique barcoded clones supplying granulocytes and B cells over time in early (n=4) and delayed aging (n=6) mice. Note that data at month 15 were collected through perfusion of all peripheral blood cells, therefore showing increased clone number. One-way repeated measures ANOVA comparing all time points; (**K**) Number of barcoded clones detected in HSCs and immune cells (granulocytes and B cells) in the peripheral blood at the end time point. (**I,J**) Each marker indicates one mouse, Horizontal bar denotes the mean of all mice. Shown are mice with available clonal data at all the indicated time points. (**A-D,F,H**) Data are mean ± SEM. WBC, white blood cells, MNC, mononuclear cells.

Supplementary Figure 6

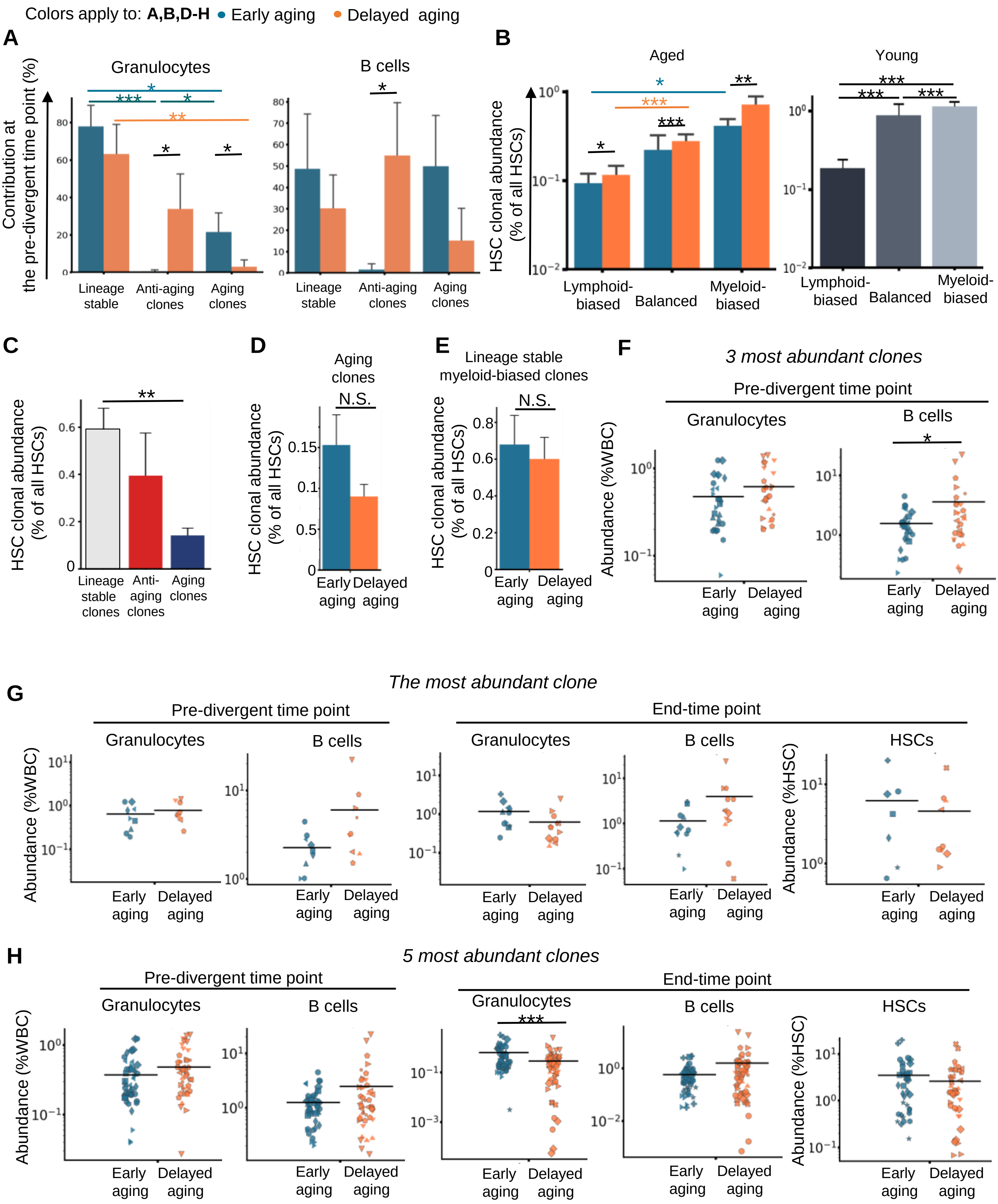

Supplementary Figure 6. Characterizing lineage stable and lineage shifting clones and clonal expansion during aging. (**A**), Contribution of lineage stable and lineage-shifting clones to granulocytes and B cells of the peripheral blood 9 months post-transplantation. One-way ANOVA followed by the Tukey HSD pairwise comparison is used for comparisons within a group; Bonferroni adjusted independent Student's t-test is used for comparisons between groups. (**B**), Average abundance of HSC clones with distinct lineage bias and balance at the end-time point and in young mice 6 months post-transplantation. (**C**) Average HSC abundance of lineage stable, anti-aging and aging clones in all mice at the end-time point. (**D**) Average HSC abundance of aging HSC clones in early and delayed aging mice. (**E**) Average HSC abundance of lineage stable myeloid-biased HSC clones in early and delayed aging mice. (**F**) Abundances of the top three most abundant clones of each mouse at the pre-divergent time point. (**G**) Abundances of the most abundant clones of each mouse. (**H**) Abundances of the top five most abundant clones of each mouse. (**F-H**) Each marker indicates one clone, and a distinct shape of markers represent clones from the same mouse. Each horizontal black bar denotes the mean of all the clones in each group. Independent Student's t-test. (**B-E**) Wilcoxon rank-sum test. Bonferroni correction is applied to (**C**). Data are mean + SEM. *P<0.05, **P<0.01, ***P<0.001. N.S., not significant. WBC, white blood cells.
